# Association of Remote Patient Monitoring with Mortality and Healthcare Utilization in Hypertensive Patients: a Medicare Claims–Based Study

**DOI:** 10.1007/s11606-023-08511-x

**Published:** 2023-11-16

**Authors:** Mahip Acharya, Mir M. Ali, Cari A. Bogulski, Ambrish A. Pandit, Ruchira V. Mahashabde, Hari Eswaran, Corey J. Hayes

**Affiliations:** 1 Institute for Digital Health & Innovation, University of Arkansas for Medical Sciences, Little Rock, AR USA; 2 Department of Biomedical Informatics, University of Arkansas for Medical Sciences, Little Rock, AR USA; 3 Divison of Pharmaceutical Evaluation and Policy, University of Arkansas for Medical Sciences, Little Rock, AR USA; 4 Center for Mental Healthcare and Outcomes Research, Central Arkansas Veterans Healthcare Systems, North Little Rock, AR USA

**Keywords:** remote patient monitoring, hypertension, medicare, mortality, healthcare utilization.

## Abstract

**Background:**

Hypertension management is complex in older adults. Recent advances in remote patient monitoring (RPM) have warranted evaluation of RPM use and patient outcomes.

**Objective:**

To study associations of RPM use with mortality and healthcare utilization measures of hospitalizations, emergency department (ED) utilization, and outpatient visits.

**Design:**

A retrospective cohort study.

**Patients:**

Medicare beneficiaries aged ≥65 years with an outpatient hypertension diagnosis between July 2018 and September 2020. The first date of RPM use with a corresponding hypertension diagnosis was recorded (index date). RPM non-users were documented from those with an outpatient hypertension diagnosis; a random visit was selected as the index date. Six months prior continuous enrollment was required.

**Main Measures:**

Outcomes studied within 180 days of index date included (i) all-cause mortality, (ii) any hospitalization, (iii) cardiovascular-related hospitalization, (iv) non-cardiovascular-related hospitalization, (v) any ED, (vi) cardiovascular-related ED, (vii) non-cardiovascular-related ED, (viii) any outpatient, (ix) cardiovascular-related outpatient, and (x) non-cardiovascular-related outpatient. Patient demographics and clinical variables were collected from baseline and index date. Propensity score matching (1:4) and Cox regression were performed. Hazard ratios (HR) and 95% confidence intervals (CI) are reported.

**Key Results:**

The matched sample had 16,339 and 63,333 users and non-users, respectively. Cumulative incidences of mortality outcome were 2.9% (RPM) and 4.3% (non-RPM), with a HR (95% CI) of 0.66 (0.60–0.74). RPM users had lower hazards of any [0.78 (0.75–0.82)], cardiovascular-related [0.79 (0.73–0.87)], and non-cardiovascular-related [0.79 (0.75–0.83)] hospitalizations. No significant association was observed between RPM use and the three ED measures. RPM users had higher hazards of any [1.10 (1.08–1.11)] and cardiovascular-related outpatient visits [2.17 (2.13–2.19)], while a slightly lower hazard of non-cardiovascular-related outpatient visits [0.94 (0.93–0.96)].

**Conclusions:**

RPM use was associated with substantial reductions in hazards of mortality and hospitalization outcomes with an increase in cardiovascular-related outpatient visits.

**Supplementary Information:**

The online version contains supplementary material available at 10.1007/s11606-023-08511-x.

## INTRODUCTION

Despite recent advances in hypertension management and treatment,^1, 2^ percentages of hypertensive adults who had their hypertension under control (systolic, <140 mmHg and diastolic, <90 mmHg) decreased from 54% in 2013–2014 to 44% in 2017–2018 in the USA.^3^ Hypertension management, particularly in older adults, is complex owing to altered pathophysiology of hypertension, presence of comorbidities, and polypharmacy.^4^ The proportion of hypertensive older adults with home-based blood pressure (BP) monitoring is as low as 14%.^5^ Regular self-measurement and documentation of BP could be an additional burden on such patients.^6^ Remote patient monitoring (RPM) is an approach in which patient data are collected outside of a healthcare setting and transmitted digitally to the providers.^7^ With advances in medical devices, cloud storage, artificial intelligence, and data sharing, RPM for vital signs such as BP has become feasible.^8–11^ Also, RPM has been combined with -omics data to personalize therapy to patients with cardiovascular diseases.^12–16^

In 2019, the Centers for Medicare & Medicaid Services (CMS) added new procedure codes for coverage and reimbursement for remote monitoring of physiological data for patients with chronic conditions.^17^ Recent revisions in 2021 and 2022 have further expanded patient eligibility and reimbursement.^18–20^ The coronavirus disease-19 (COVID-19) pandemic has spurred the adoption of these services: a recent study in fee-for-service Medicare population reported an approximately 600% increase in RPM use from February 2020 to September 2021.^21^ The study found hypertension diagnosis in 63% of RPM claims.^21^

Understanding whether RPM for BP management is effective and to what magnitude, if any, is crucial to expanding RPM infrastructure and adopting these services in clinical practice.^22^ Although clinical trials have been conducted for RPM, the studies are limited by small sample size and heterogeneity in the interventions and control groups across studies.^23^ To this end, we conducted a cohort study among older adults with hypertension to estimate the relationships between RPM use and all-cause mortality, hospitalizations, emergency department (ED), and outpatient visits.

## METHODS

### Data

A 20% random sample of fee-for-service Medicare beneficiaries (2018–2020) was used. The Area Health Resource File (2019–2020), a publicly available dataset, was used to add county-level population demographics and healthcare utilization.^24^

### Study Design and Population

We conducted a retrospective cohort study of Medicare beneficiaries diagnosed with hypertension. RPM users were identified between July 2018 and December 2020. The first RPM claim with a corresponding hypertension diagnosis was considered as the index date. RPM non-users were identified from individuals with an outpatient hypertension diagnosis between July 2018 and December 2020 but without any RPM claims during this timeframe. For each RPM non-user, a random hypertension visit was selected from their pool of hypertension-related outpatient visits (index date) in July 2018–Dec 2020 period. Both RPM users and non-users were required to have continuous enrollment in Medicare Parts A and B during the 6 months prior (baseline period). Beneficiaries <65 years or >100 years of age on index date were excluded. The sample was restricted to individuals with index dates between July 2018 and September 2020 to allow for some follow-up time for assessing outcomes, as the data ended in December 2020. The cohort was followed from the index date until the outcome of interest (evaluated separately for each outcome), loss of enrollment, data end date, or a maximum of 180 days, whichever date was earliest. The manuscript was determined to be human subjects research by the University of Arkansas for Medical Sciences (UAMS) Institutional Review Board (IRB) and was approved under IRB #263124. We followed the STROBE reporting guideline for cohort studies.

### Exposure

RPM use for hypertension was the exposure of interest. RPM was identified using Current Procedural Technology (CPT) codes and CPT modifiers (Supplement Table 1). Essential hypertension was identified using International Classification of Diseases-10-Clinical Modification (ICD-10-CM) diagnosis code of I10 (Supplement Table 1). The data do not have blood pressure readings, but the diagnosis code generally corresponds to systolic BP ≥140 mmHg and diastolic BP ≥90 mmHg.^25^ The index visit for RPM non-users was selected using random sampling.

### Outcomes

The outcomes studied were (i) all-cause mortality, (ii) any hospitalization, (iii) cardiovascular-related hospitalization, (iv) non-cardiovascular-related hospitalization, (v) any emergency department (ED) visit, (vi) cardiovascular-related ED visit, (vii) non-cardiovascular-related ED visit, (viii) any outpatient visit, (ix) cardiovascular-related outpatient visit, and (x) non-cardiovascular-related outpatient visit. All-cause mortality was identified using the date of death information. Hospitalizations with diagnosis codes for cardiovascular conditions as the admission or primary diagnosis were considered as cardiovascular-related hospitalizations (Supplement Table 1). We used a combination of prior literature and our clinical knowledge to define cardiovascular-related hospitalizations a priori.^26, 27^ The time to first cardiovascular-related hospitalization or censoring was determined. We also determined non-cardiovascular-related hospitalizations by requiring the absence of previously defined cardiovascular conditions.

ED visits were identified using a combination of CPT procedure codes and a place of service code of “23.” Outpatient visits were identified using place of service codes. Cardiovascular-related and non-cardiovascular-related ED and outpatient visits were defined similar to hospitalizations.

### Covariates

Information on age, sex, and race/ethnicity was collected on index date. Rural/urban residence was identified in the index year using rural-urban commuting area (RUCA) codes.^28^ Cardiovascular and non-cardiovascular comorbidities, healthcare utilization, and prescription medications were identified in baseline. Since Part D enrollment was not required in the baseline, medication use was categorized into three groups: yes, no, and no enrollment. Pneumonia and septicemia were documented, as they are common causes of hospitalization and deaths among older adults. To capture healthcare-seeking behavior, presence of a wellness visit and flu vaccination in the baseline was determined for both RPM users and non-users. The following county-level characteristics were gathered: hospitalization rates, outpatient visit rates, poverty rates, and health professional shortage area (HPSA) designation for primary care.

### Statistical Analyses

Absolute percentage standardized differences and variance ratio were calculated for the baseline characteristics by comparing the RPM users and non-users.^29^ A propensity score model was estimated using the RPM use status as the outcome and the covariates previously described as the independent variables. A 1:4 nearest-neighbor matching on the estimated propensity score and exact matching on index year and the presence of complicated hypertension were performed between RPM users and non-users. A 1:4 matching was chosen based on prior research that has shown that using multiple controls for rare exposure (1:3 through 1:5) leads to an increase in precision.^30^ The Kaplan-Meier method was used to estimate survival curves for RPM users and non-users for all outcomes. Cox proportional hazard regressions were used to estimate relative hazards of the outcomes with RPM use as the exposure. Hazard ratios and 95% confidence intervals are reported. For the healthcare utilization outcomes (hospitalizations, ED, outpatient), competing risk Cox regressions were performed using all-cause mortality as the competing event, and cause-specific hazard ratios were estimated. All analyses were performed using SAS 9.4 and R/RStudio.

### Sensitivity Analyses

We conducted several sensitivity analyses to analyze the robustness of our primary findings. First, to allow for 30 days of RPM, we restricted the final, unmatched sample to those who survived for at least 30 days from their index date and redefined the index date as the initial index date + 30 days. We then reperformed the matching and regressions. Second, we used the final unmatched sample from the primary analysis and performed entropy balance weighting instead of matching. Third, we constructed a group of RPM non-users using a different approach: we identified hypertensive patients for each month in July 2018–September 2020 period, randomly selected 50% exclusively for each month, and defined the last hypertension visit date for the respective month as the index date. This approach was used to avoid inadvertently using an individual’s future healthcare utilization to define an index date. Fourth, we conducted an analysis where we exactly matched on index year and index month in addition to the estimated propensity score. Fifth, we used a positive control outcome of depression: a positive control outcome is the one where we expect an effect of the intervention based on prior literature and clinical rationale.^31^ A prior study has shown that RPM use worsens depression.^32^ Sixth, to test whether RPM users are systematically healthier or sicker beyond that captured by the covariates, we moved the index date to an outpatient visit between 60 and 90 days prior to the original index date. For example, if RPM users were healthier, then shifting the index date should still yield estimates indicating protective effect. We estimated the Cox models using time to any ED and any hospitalization using the new index date.

Two additional sensitivity analyses were conducted for the mortality outcome. As the database does not have cause of death, we used a proxy measure to define cardiovascular-related mortality. We recorded any healthcare visit within 30 days of the date of death for those who died during follow-up and looked for presence of any cardiovascular condition as the primary or secondary diagnosis. We modeled time to cardiovascular mortality using non-cardiovascular mortality as a competing event. In the final sensitivity analysis, we used an instrumental variable approach to account for potential unmeasured confounding between RPM use and mortality. We used calendar month as a continuous instrumental variable,^33^ using prior knowledge that RPM use had increased with time in the recent years. We used two-stage residual inclusion for estimating hazard ratios, and 500 bootstrapped samples for confidence intervals.

## RESULTS

**Figure 1:**
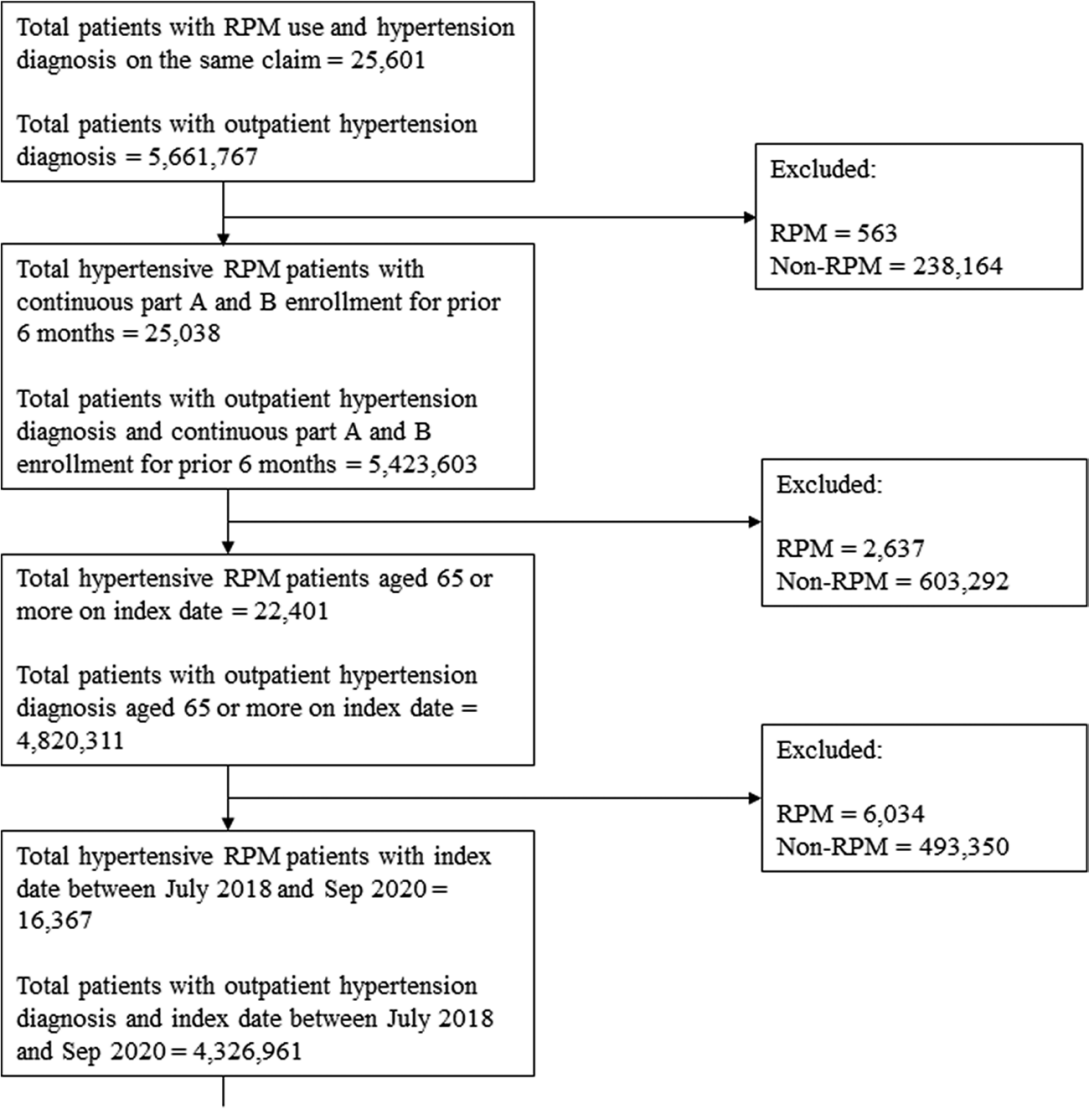

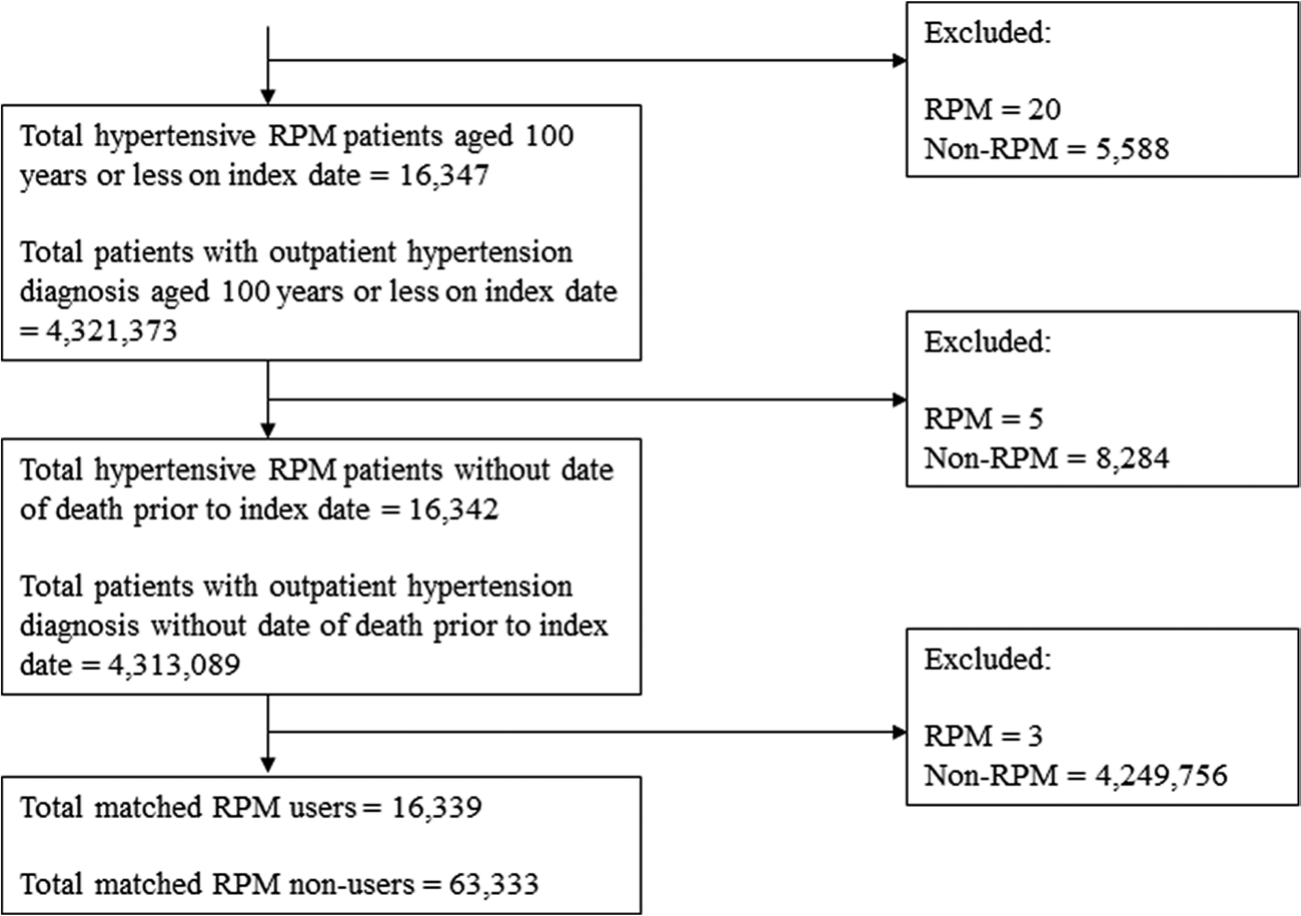
**Flow diagram of sample selection.**

A total of 25,601 and 5,661,767 hypertensive individuals with and without RPM use, respectively, were identified. After applying the restrictions of continuous enrollment, age range, and index date between July 2018 and September 2020, 16,342 RPM users and 4,313,089 RPM non-users remained in the study sample. Following the matching procedure, 16,339 RPM users and 63,333 RPM non-users were retained (Fig. 1).

The mean age was 76 years across both groups (Table 1). Approximately 3% of both groups had a history of myocardial infarction. Around 26% of RPM users had complicated hypertension, while only 18% of non-users had the condition, resulting in a standardized difference of 21%. The average number of cardiovascular-related outpatient visits was 3.5 among RPM users and 1.5 among non-users with a standardized difference of 107% (Table 1).

**Table 1:** **Baseline Characteristics of the Full Sample**

Patient characteristics	RPM users (*N* = 16,342)	RPM non-users (*N* = 4,313,089)	Absolute mean standardized differences (%)	Variance ratio
	*n* (%)	*n* (%)		
Age (years): mean (SD)	76.22 (7.45)	76.23 (7.80)	0.19	0.91
Sex				
Male	6864 (42.00)	1,898,353 (44.01)	4.06	0.99
Female	9478 (58.00)	2,414,736 (55.99)		
Race/ethnicity				
Non-Hispanic White	12,017 (73.53)	3,710,011 (86.02)	31.46	1.62
Non-Hispanic Black	2399 (14.68)	323,346 (7.50)	23.03	1.81
Hispanic	426 (2.61)	57,851 (1.34)	9.12	1.92
Asian	719 (4.40)	63,257 (1.47)	17.45	2.91
Native American	41 (0.25)	26,546 (0.62)	5.55	0.41
Other	370 (2.26)	60,523 (1.40)	6.42	1.60
Unknown	370 (2.26)	71,555 (1.66)		
Index year				
2018	391 (2.39)	1,033,291 (23.96)		
2019	3449 (21.11)	1,995,073 (46.26)	55.21	0.67
2020	12,502 (76.50)	1,284,725 (29.79)	105.94	0.86
Rural/urban classification				
Large rural	1255 (7.68)	595,649 (13.81)	19.89	0.60
Small rural	997 (6.10)	604,083 (14.01)	26.52	0.48
Urban	12,694 (77.68)	2,637,341 (61.15)	36.47	0.73
Sub-urban	1361 (8.33)	472,237 (10.95)	8.89	0.78
Missing	35 (0.21)	3779 (0.09)		
Comorbidity measures				
Complicated hypertension	4325 (26.47)	759,619 (17.61)	21.48	1.34
Myocardial infarction	414 (2.53)	108,885 (2.52)	0.06	1.00
Congestive heart failure	3411 (20.87)	645,594 (14.97)	15.44	1.30
Stroke	1374 (8.41)	301,671 (6.99)	5.30	1.18
Acute renal failure	3960 (24.23)	759,118 (17.60)	16.36	1.27
Alcohol use disorder	303 (1.85)	78,930 (1.83)	0.18	1.01
Iron deficiency anemia	2420 (14.81)	397,781 (9.22)	17.24	1.51
Cardiac arrhythmia	5265 (32.22)	1,333,211 (30.91)	2.81	1.02
Valvular disease	2887 (17.67)	680,312 (15.77)	5.07	1.09
Blood loss anemia	413 (2.53)	84,751 (1.96)	3.80	1.28
Chronic pulmonary disease	4074 (24.93)	954,076 (22.12)	6.63	1.09
Circulation disorders	838 (5.13)	203,688 (4.72)	1.87	1.08
Coagulopathy	753 (4.61)	198,514 (4.60)	0.03	1.00
Depression	3289 (20.13)	750,726 (17.41)	6.97	1.12
Drug abuse	600 (3.67)	69,555 (1.61)	12.86	2.23
Uncomplicated diabetes	5915 (36.20)	1,297,484 (30.08)	13.01	1.10
Complicated diabetes	4845 (29.65)	896,782 (20.79)	20.50	1.27
Fluid and electrolyte disorders	2474 (15.14)	662,085 (15.35)	0.59	0.99
HIV/AIDS	23 (0.14)	6137 (0.14)	0.04	0.99
Hypothyroidism	4455 (27.26)	1,017,781 (23.60)	8.42	1.10
Liver disease	965 (5.91)	232,798 (5.40)	2.20	1.09
Lymphoma	207 (1.27)	61,726 (1.43)	1.43	0.89
Solid tumors (excluding metastasis)	1933 (11.83)	572,881 (13.28)	4.39	0.91
Metastasis	242 (1.48)	113,531 (2.63)	8.12	0.57
Other neurological disorders	1620 (9.91)	407,147 (9.44)	1.60	1.04
Obesity	3653 (22.35)	717,943 (16.65)	14.44	1.25
Weight loss	789 (4.83)	217,345 (5.04)	0.98	0.96
Paralysis	328 (2.01)	61,870 (1.43)	4.40	1.39
Peptic ulcer disease	187 (1.14)	56,649 (1.31)	1.54	0.87
Peripheral vascular disorders	4590 (28.09)	860,582 (19.95)	19.13	1.26
Psychoses	219 (1.34)	59,686 (1.38)	0.38	0.97
Pneumonia	904 (5.53)	234,188 (5.43)	0.45	1.02
Rheumatoid arthritis	1398 (8.55)	285,098 (6.61)	7.35	1.27
Septicemia	475 (2.91)	129,787 (3.01)	0.61	0.97
Health behavior measures				
Annual wellness visit	4585 (28.06)	722,305 (16.75)	27.38	1.45
Flu vaccination	3234 (19.79)	1,008,859 (23.39)	8.76	0.89
Chronic care management	6111 (37.39)	111,897 (2.59)	96.63	9.26
Prescription measures				
Part D enrollment	12,274 (75.11)	3,084,278 (71.51)	8.14	0.92
Antihypertensives				
Yes	11,371 (69.58)	2,817,090 (65.31)	9.12	0.93
No	1035 (6.33)	307,350 (7.13)	3.16	0.90
No Part D enrollment	3936 (24.09)	1,188,649 (27.56)		
Antithrombotics				
Yes	3419 (20.92)	745,513 (17.28)	9.26	1.16
No	8894 (54.42)	2,348,844 (54.46)	0.07	1.00
No Part D enrollment	4029 (24.65)	1,218,732 (28.26)		
Antihyperglycemics				
Yes	3666 (22.43)	764,740 (17.73)	11.76	1.19
No	8650 (52.93)	2,330,853 (54.04)	2.23	1.00
No Part D enrollment	4026 (24.64)	1,217,496 (28.23)		
Antiarrhythmics				
Yes	732 (4.48)	179,464 (4.16)	1.57	1.07
No	11,547 (70.66)	2,907,018 (67.40)	7.05	0.94
No Part D enrollment	4063 (24.86)	1,226,607 (28.44)		
Antihyperlipidemics				
Yes	8038 (49.19)	1,885,016 (43.70)	11.01	1.02
No	4312 (26.39)	1,222,984 (28.36)	4.42	0.96
No Part D enrollment	3992 (24.43)	1,205,089 (27.94)		
Healthcare utilization measures	Mean (SD)	Mean (SD)		
Total medical cost	10,153.27 (20,122.07)	10,481.13 (21,307.65)	1.58	0.89
Total ED visits	0.40 (0.93)	0.64 (1.12)	23.59	0.68
Total hospitalizations	0.21 (0.63)	0.23 (0.64)	2.73	0.97
Total outpatient visits	12.23 (9.17)	8.84 (8.16)	38.95	1.26
Cardiovascular-related ED visits	0.06 (0.30)	0.07 (0.31)	2.32	1.01
Cardiovascular-related hospitalizations	0.21 (0.62)	0.22 (0.62)	6.03	0.82
Cardiovascular-related outpatient visits	3.53 (2.99)	1.45 (2.08)	107.32	3.60
Area-level characteristics	*n* (%)	*n* (%)		
HPSA categories				
Full shortage	774 (4.74)	294,955 (6.84)	9.01	0.71
Partial shortage	14,714 (90.04)	3,600,727 (83.48)	19.43	0.65
No shortage	828 (5.07)	411,448 (9.54)	17.26	0.56
Missing	26 (0.16)	5959 (0.14)		
County-level total hospitalization rates				
First quartile	2968 (18.16)	1,076,556 (24.96)	16.59	0.79
Second quartile	4247 (25.99)	1,082,794 (25.10)	2.03	1.02
Third quartile	5920 (36.23)	1,068,119 (24.76)	25.09	1.24
Fourth quartile	3150 (19.28)	1,077,795 (24.99)	13.80	0.83
Missing	57 (0.35)	7825 (0.18)		
County-level outpatient visit rates				
First quartile	5175 (31.67)	1,079,712 (25.03)	14.76	1.15
Second quartile	5329 (32.61)	1,071,655 (24.85)	17.22	1.18
Third quartile	3377 (20.66)	1,074,014 (24.90)	10.11	0.88
Fourth quartile	2404 (14.71)	1,079,883 (25.04)	26.10	0.67
Missing	57 (0.35)	7825 (0.18)		
County-level poverty rates				
First quartile	3650 (22.34)	1,085,687 (25.17)	6.67	0.92
Second quartile	3722 (22.78)	1,066,353 (24.72)	4.58	0.95
Third quartile	4466 (27.33)	1,093,404 (25.35)	4.49	1.05
Fourth quartile	4444 (27.19)	1,056,345 (24.49)	6.18	1.07
Missing	60 (0.37)	11,300 (0.26)		

*HPSA*, health professional shortage area

Five categories were created using the continuous RUCA codes: urban (1), sub-urban (2, 3), large rural (4–6), small rural (7–10), and missing

Baseline characteristics were balanced between the matched groups, as indicated by standardized differences of lower than 10% for all variables (Table 2). In the matched sample, the average number of outpatient visits among RPM users and non-users was 12.2 and 12.0 respectively, while the averages for cardiovascular-related outpatient visits were 3.5 (RPM users) and 3.2 (non-users). The distributions of propensity scores are shown in Supplement Figures 1 and 2.

**Table 2:** **Baseline Characteristics of the Matched Sample**

Patient characteristics	RPM users (*n* = 16,339)	RPM non-users (*n* = 63,333)	Absolute mean standardized differences (%)	Variance ratio
	*n* (%)	*n* (%)		
Age (years): mean (SD)	76.22 (7.46)	76.26 (7.57)	0.04	1.00
Sex			1.13	1.00
Male	6862 (42.00)	26,275 (41.49)		
Female	9477 (58.00)	37,058 (58.51)		
Race/ethnicity				
Non-Hispanic White	12,017 (73.55)	46,063 (72.73)	3.68	0.97
Non-Hispanic Black	2398 (14.68)	9806 (15.48)	3.60	0.94
Hispanic	426 (2.61)	1660 (2.62)	0.77	0.96
Asian	717 (4.39)	2714 (4.29)	0.60	0.98
Native American	41 (0.25)	141 (0.22)	0.47	1.14
Other	370 (2.26)	1475 (2.33)	0.67	0.96
Unknown	370 (2.26)	1474 (2.33)		
Index year				
2018	391 (2.39)	1564 (2.47)		
2019	3449 (21.11)	13,794 (21.78)	0.00	1.00
2020	12,499 (76.50)	47,975 (75.75)	0.00	1.00
Rural/urban classification				
Large rural	1255 (7.68)	4524 (7.14)	2.20	1.09
Small rural	997 (6.10)	3537 (5.58)	2.19	1.11
Urban	12,691 (77.67)	50,038 (79.01)	3.74	1.06
Sub-urban	1361 (8.33)	5078 (8.02)	1.38	1.05
Missing	35 (0.21)	156 (0.25)		
Comorbidity measures				
Complicated hypertension	4324 (26.46)	16,859 (26.62)	0.00	1.00
Myocardial infarction	414 (2.53)	1589 (2.51)	0.22	1.01
Congestive heart failure	3409 (20.86)	13,296 (20.99)	1.01	0.99
Stroke	1374 (8.41)	5385 (8.50)	0.82	0.98
Acute renal failure	3958 (24.22)	15,780 (24.92)	1.94	0.98
Alcohol use disorder	303 (1.85)	1156 (1.83)	0.08	1.01
Iron deficiency anemia	2418 (14.80)	9545 (15.07)	1.81	0.97
Cardiac arrhythmia	5264 (32.22)	20,803 (32.85)	1.92	0.99
Valvular disease	2887 (17.67)	11,443 (18.07)	1.40	0.98
Blood loss anemia	413 (2.53)	1700 (2.68)	1.21	0.94
Chronic pulmonary disease	4072 (24.92)	15,867 (25.05)	0.75	0.99
Circulation disorders	838 (5.13)	3340 (5.27)	0.71	0.97
Coagulopathy	752 (4.60)	2994 (4.73)	0.78	0.97
Depression	3289 (20.13)	12,921 (20.40)	1.24	0.98
Drug abuse	600 (3.67)	2371 (3.74)	1.62	0.94
Uncomplicated diabetes	5913 (36.19)	23,031 (36.36)	0.97	0.99
Complicated diabetes	4843 (29.64)	18,834 (29.74)	1.12	0.99
Fluid and electrolyte disorders	2473 (15.14)	9915 (15.66)	1.43	0.97
HIV/AIDS	23 (0.14)	84 (0.13)	0.26	1.07
Hypothyroidism	4454 (27.26)	17,496 (27.63)	1.20	0.99
Liver disease	965 (5.91)	3858 (6.09)	0.89	0.97
Lymphoma	206 (1.26)	814 (1.29)	0.14	0.99
Solid tumors (excluding metastasis)	1933 (11.83)	7513 (11.86)	0.06	1.00
Metastasis	242 (1.48)	1001 (1.58)	0.51	0.95
Other neurological disorders	1620 (9.91)	6459 (10.20)	1.19	0.97
Obesity	3653 (22.36)	14,243 (22.49)	0.86	0.99
Weight loss	789 (4.83)	3237 (5.11)	1.49	0.94
Paralysis	328 (2.01)	1277 (2.02)	0.46	0.97
Peptic ulcer disease	187 (1.14)	770 (1.22)	0.73	0.94
Peripheral vascular disorders	4588 (28.08)	17,762 (28.05)	1.14	0.99
Psychoses	219 (1.34)	883 (1.39)	0.49	0.96
Pneumonia	904 (5.53)	3652 (5.77)	1.04	0.96
Rheumatoid arthritis	1398 (8.56)	5545 (8.76)	1.26	0.97
Septicemia	475 (2.91)	2011 (3.18)	1.45	0.92
Health behavior measures				
Annual wellness visit	4583 (28.05)	17,994 (28.41)	2.30	0.98
Flu vaccination	3234 (19.79)	12,379 (19.55)	0.76	1.01
Chronic care management	6108 (37.38)	22,338 (35.27)	0.66	1.00
Prescription measures				
Part D enrollment	12,272 (75.11)	47,577 (75.12)	0.37	1.00
Antihypertensives				
Yes	11,369 (69.58)	44,103 (69.64)	0.55	1.00
No	1035 (6.33)	4020 (6.35)	0.16	1.01
No enrollment	3935 (24.08)	15,210 (24.02)		
Antithrombotics				
Yes	3418 (20.92)	13,212 (20.86)	0.71	0.99
No	8893 (54.43)	34,523 (54.51)	0.22	1.00
No enrollment	4028 (24.65)	15,598 (24.63)		
Antihyperglycemics				
Yes	3664 (22.42)	14,125 (22.30)	0.17	1.00
No	8650 (52.94)	33,634 (53.11)	0.26	1.00
No enrollment	4025 (24.63)	15,574 (24.59)		
Antiarrhythmics				
Yes	732 (4.48)	2860 (4.52)	0.45	0.98
No	11,545 (70.66)	44,736 (70.64)	0.16	1.00
No enrollment	4062 (24.86)	15,737 (24.85)		
Antihyperlipidemics				
Yes	8037 (49.19)	30,999 (48.95)	0.04	1.00
No	4311 (26.38)	16,866 (26.63)	0.29	1.00
No enrollment	3991 (24.43)	15,468 (24.42)		
Healthcare utilization measures	Mean (SD)	Mean (SD)		
Total medical cost	10,147.18 (20,103.91)	10,579.86 (18,598.47)	2.19	1.18
Total ED visits	0.40 (0.93)	0.42 (0.79)	1.57	1.39
Total hospitalizations	0.21 (0.63)	0.23 (0.62)	2.15	1.04
Total outpatient visits	12.22 (9.17)	11.95 (9.60)	0.05	0.88
Cardiovascular-related ED visits	0.06 (0.30)	0.06 (0.27)	0.29	1.18
Cardiovascular-related hospitalizations	0.21 (0.62)	0.22 (0.60)	2.05	1.06
Cardiovascular-related outpatient visits	3.53 (2.98)	3.15 (3.87)	7.06	0.51
HPSA categories for primary care				
Full shortage	774 (4.74)	3261 (5.15)	1.67	0.93
Partial shortage	14,711 (90.04)	55,053 (86.93)	8.86	0.80
No shortage	828 (5.07)	4883 (7.71)	9.81	0.68
Missing	26 (0.16)	136 (0.21)		
County-level total hospitalization rates				
First quartile	2967 (18.16)	10,980 (17.34)	2.52	1.05
Second quartile	4247 (25.99)	16,513 (26.07)	0.14	1.00
Third quartile	5918 (36.22)	23,505 (37.11)	2.96	0.98
Fourth quartile	3150 (19.28)	12,099 (19.10)	1.00	1.02
Missing	57 (0.35)	236 (0.37)		
County-level outpatient visit rates				
First quartile	5174 (31.67)	20,055 (31.67)	0.53	1.00
Second quartile	5327 (32.60)	21,117 (33.34)	2.13	0.99
Third quartile	3377 (20.67)	13,076 (20.65)	0.58	1.01
Fourth quartile	2404 (14.71)	8849 (13.97)	2.52	1.06
Missing	57 (0.35)	236 (0.37)	1.25	1.02
County-level poverty rates				
First quartile	3650 (22.34)	14,008 (22.12)	1.25	1.02
Second quartile	3721 (22.77)	14,475 (22.86)	0.21	1.00
Third quartile	4464 (27.32)	17,225 (27.20)	0.04	1.00
Fourth quartile	4444 (27.20)	17,372 (27.43)	0.94	0.99
Missing	60 (0.37)	253 (0.40)		

*HPSA*, health professional shortage area

Five categories were created using the continuous RUCA codes: urban (1), sub-urban (2, 3), large rural (4–6), small rural (7–10), and missing

The average follow-up was 163 days for both RPM users and non-users for all-cause mortality (Table 3). The rates of any hospitalizations were 8.16 (users) and 10.46 (non-users) per 10,000 person-days. The distributions of patients with multiple outcomes are shown in Supplement Table 2.

**Table 3:** **Proportion and Rates of the Outcomes Across RPM Users and Non-users in the Primary Analysis (Matched Sample)**

Outcomes	RPM users (*n* = 16,339)			RPM non-users (*n* = 63,333)		
	Events; *n* (%)	Total follow-up (mean follow-up) (days)	Event rate (events per 10,000)	Events; *n* (%)	Total follow-up (mean follow-up) (days)	Event rate (events per 10,000)
All-cause mortality	430 (2.63)	2,655,777 (163)	1.62	2519 (3.98)	10,332,260 (163)	2.44
Any hospitalization	2026 (12.40)	2,482,948 (152)	8.16	9837 (15.53)	9,407,052 (149)	10.46
Cardiovascular-related hospitalization	576 (3.53)	2,607,796 (160)	2.21	2800 (4.42)	10,080,646 (159)	2.78
Non-cardiovascular-related hospitalization	1652 (10.11)	2,519,370 (154)	6.56	7982 (12.60)	9,598,852 (152)	8.32
Any ED visit	3333 (20.40)	2,355,854 (144)	14.15	13,373 (21.12)	9,092,793 (144)	14.71
Cardiovascular-related ED visit	584 (3.57)	2,606,651 (160)	2.24	2345 (3.70)	10,137,701 (160)	2.31
Non-cardiovascular-related ED visit	3042 (18.62)	2,385,146 (146)	12.75	12,183 (19.24)	9,211,306 (145)	13.23
Any outpatient visit	15,924 (97.46)	320,010 (20)	497.61	59,572 (94.06)	1,663,325 (26)	358.15
Cardiovascular-related outpatient visit	14,725 (90.12)	670,297 (41)	219.68	40,503 (63.95)	5,392,499 (85)	75.11
Non-cardiovascular-related outpatient visit	15,362 (94.02)	551,805 (34)	278.40	58,350 (92.13)	2,067,888 (33)	282.17

**Figure 2:**
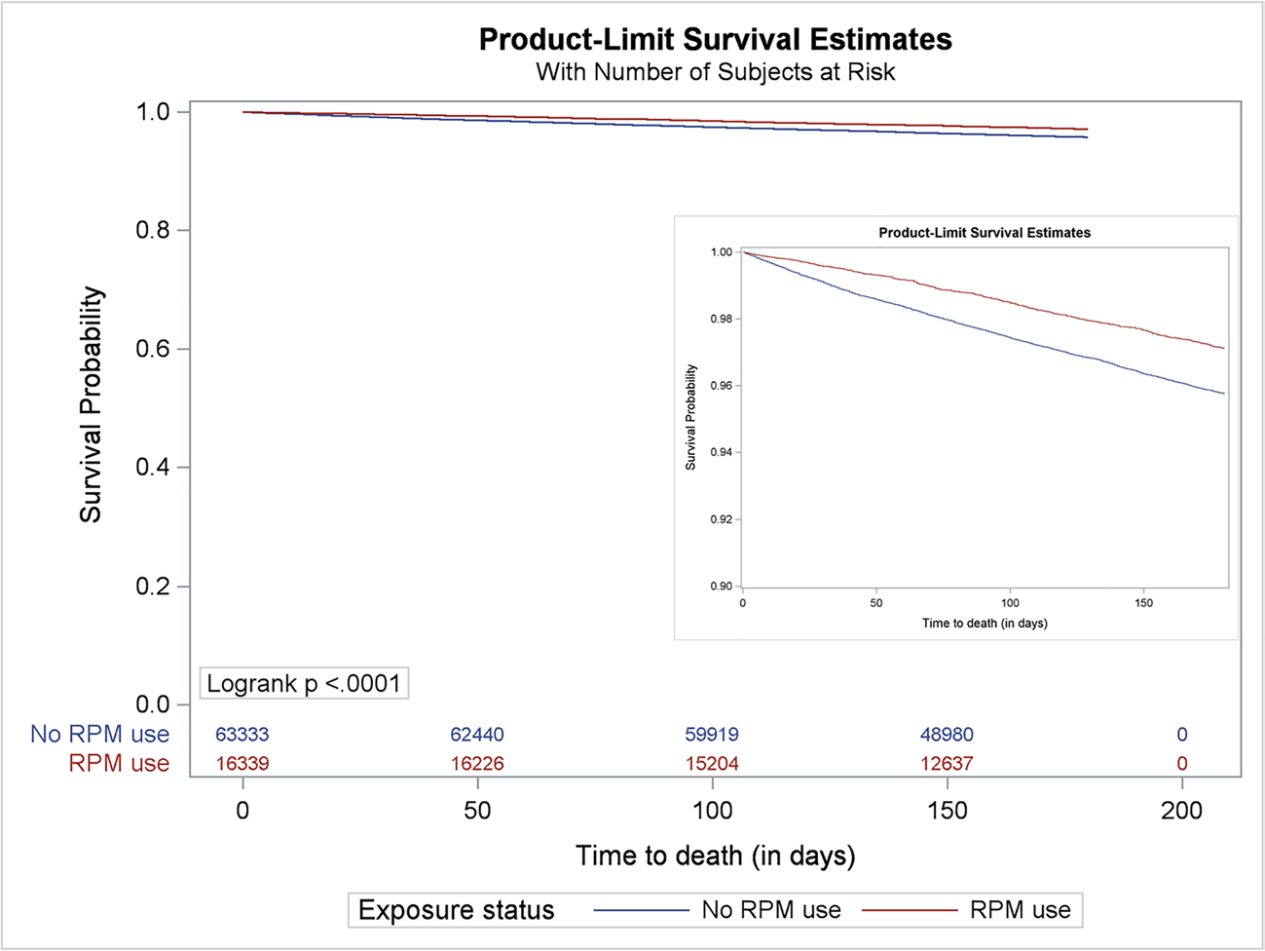
Kaplan-Meier curves for all-cause mortality outcome in the matched sample in the primary analysis. Note: The inset chart is included to increase visibility and represents the same Kaplan-Meier curves for all-cause mortality outcome with the *Y*-axis beginning at 0.90 survival probability.

Kaplan-Meier analysis in the matched sample showed that the cumulative incidences of mortality were 2.9% (RPM users) and 4.3% (non-users) (*p* < 0.001; Fig. 2). In the primary analysis, RPM users had a lower hazard of mortality compared to RPM non-users [HR, 0.66 (0.60–0.74)]. Kaplan-Meier curves for healthcare utilization outcomes are shown in Supplement Figures 3–11. RPM users had lower rates of any hospitalizations [HR, 0.78 (0.75–0.82)], cardiovascular-related hospitalizations [HR, 0.79 (0.73–0.87)], and non-cardiovascular-related hospitalizations [HR, 0.79 (0.75–0.83)] (Table 4). No significant association was observed with any of the three ED outcomes. Hazard ratios for any outpatient visit, cardiovascular-related outpatient visit, and non-cardiovascular-related outpatient visit were 1.10 (1.08–1.11), 2.17 (2.13–2.19), and 0.94 (0.93–0.96) respectively.

**Table 4:** **Cox Proportional Hazard Regression Results (Reference=No RPM Use)**

Outcomes	Main analysis	30-day duration	Entropy weight balancing	Alternative approach for control group	Matching on both index year and month
All-cause mortality	0.66 (0.60–0.74)	0.72 (0.65–0.80)	0.63 (0.56–0.72)	0.75 (0.67–0.83)	0.64 (0.58–0.71)
Any hospitalization	0.78 (0.75–0.82)	0.90 (0.86–0.94)	0.77 (0.74–0.81)	0.73 (0.69–0.76)	0.76 (0.73–0.80)
Cardiovascular-related hospitalization	0.79 (0.73–0.87)	0.89 (0.81–0.98)	0.77 (0.71–0.83)	0.76 (0.69–0.83)	0.78 (0.72–0.86)
Non-cardiovascular-related hospitalization	0.79 (0.75–0.83)	0.91 (0.86–0.96)	0.79 (0.75–0.82)	0.73 (0.69–0.77)	0.77 (0.73–0.81)
Any ED visit	0.96 (0.93–1.00)	1.05 (1.01–1.09)	0.97 (0.94–1.01)	0.90 (0.87–0.94)	0.96 (0.93–1.00)
Cardiovascular-related ED visit	0.97 (0.89–1.06)	1.08 (0.99–1.19)	0.98 (0.91–1.07)	0.91 (0.84–1.00)	0.97 (0.88–1.07)
Non-cardiovascular-related ED visit	0.96 (0.93–1.00)	1.05 (1.00–1.09)	0.98 (0.94–1.01)	0.91 (0.87–0.94)	0.97 (0.93–1.01)
Any outpatient visit	1.10 (1.08–1.11)	1.68 (1.65–1.72)	1.09 (1.07–1.11)	1.10 (1.08–1.12)	1.09 (1.08–1.11)
Cardiovascular-related outpatient visit	2.17 (2.13–2.19)	2.91 (2.85–2.98)	2.15 (2.11–2.18)	2.22 (2.18–2.26)	2.20 (2.17–2.26)
Non-cardiovascular-related outpatient visit	0.94 (0.93–0.96)	1.13 (1.11–1.15)	0.94 (0.93–0.96)	0.94 (0.93–0.96)	0.94 (0.92–0.95)
Additional sensitivity analyses for mortality outcome					
Cardiovascular-related mortality	0.72 (0.60–0.87)				
Instrumental variable analysis for all-cause mortality	0.56 (0.50–0.61)				
Propensity score matched analysis for depression outcome	1.19 (1.11–1.27)				
Analyses where index date was shifted to an outpatient visit between 60 and 90 days prior to the index date					
Any hospitalization	1.08 (1.02–1.14)				
Any ED visit	1.21 (1.16–1.27)				

The analysis for cardiovascular-related mortality showed a 28% reduction [HR, 0.72 (0.60–0.87)] with RPM use. In the instrumental variable sensitivity analysis, RPM use was associated with a 44% lower hazard of all-cause mortality [HR, 0.56 (0.50–0.61)]. The results of other sensitivity analyses were consistent with the primary analysis except for ED outcomes: RPM use was associated with a modest increase in any ED visit in 30-day duration analysis [HR, 1.05 (1.01–1.09)] but was associated with lower hazard of any ED visit [HR, 0.90 (0.87–0.94)] in the alternative control group approach (Table 4). The analyses with index date shifted to 60–90 days showed higher hazard of hospitalization and ED visits among RPM users, suggesting that RPM users may not be systematically healthier.

## DISCUSSION

To the best of our knowledge, this is the first population-level study to assess associations between RPM use and mortality and healthcare utilization rates. We found that RPM use in hypertensive patients is associated with reductions in all-cause mortality, cardiovascular-related mortality, all-cause hospitalizations, and cardiovascular-related hospitalizations. There were some inconsistent findings regarding ED visits, while RPM users had higher cardiovascular-related outpatient visits and slightly lower non-cardiovascular-related outpatient visits than RPM non-users.

Most other studies of RPM have evaluated intermediate outcomes. One randomized trial compared three treatment groups—RPM with office visits, RPM with remote care, and ambulatory self-measurement with office visits—and found that the two groups with RPM had better BP control (69% and 67%) compared to the routine care group (50%) at 24 weeks of follow-up among patients >55 years of age.^34^ Likewise, a meta-analysis that compared telemonitoring and routine care reported an average decrease of 7 mmHg for systolic BP in the telemonitoring group compared to 3 mmHg in the control group.^35^ Our study assessed clinical outcomes such as mortality and hospitalizations, and found consistent results regarding the superiority of RPM for BP management.

A randomized trial published in 2020, however, reported no difference in mortality and hospitalization between patients randomized to RPM and those to routine care.^36^ However, the study was not powered for mortality and hospitalization outcomes (100 patients in each group), and the median age of the participants was 60 (range, 28–61).^36^ This could explain the inconsistency with our findings. All-cause mortality in that study was 2% in each of the treatment groups at 1 year of follow-up,^36^ unlike ours with 3% (RPM) and 4% (non-RPM) over 6 months, which suggests different patient comorbid profiles. In another study, the addition of remote blood pressure and weight monitoring for patients with heart failure who are already remotely monitored for cardiac rhythm using implantable defibrillators did not reduce the risk of mortality and hospitalizations.^37^ This raises questions about how and where RPM fits in for patients with multiple comorbid conditions, and future studies are required to tease out incremental value of different RPM modalities.

We found that the reduction in hazard for RPM users for the all-cause mortality outcome was greater than that for hospitalization. A 2019 study reported that older adults in the USA are increasingly dying at home with a steady decline in the proportion of deaths in hospital settings.^38^ Cardiovascular disease was also responsible for the highest proportion of deaths (29%).^38^ In our sensitivity analysis for cardiovascular-related mortality, we found that only 27% of the deaths had a healthcare claim in the 30-day window prior to death. Some evidence exists regarding benefits of telemonitoring of hypertensive patients on improved adherence to antihypertensive medications,^39^ although the evidence base is mixed.^34, 40^ Also, prior studies have reported meaningful benefits of antihypertensive medication adherence on hospitalizations and mortality.^41, 42^ These previous findings indicate that the more pronounced benefits of RPM use for mortality could likely be mediated through better adherence to medications and other chronic disease management behaviors. This could also explain the similar associations of RPM use with cardiovascular-related and non-related hospitalizations.

Our analyses for ED visits yielded inconsistent findings, demonstrating that effect of RPM use on ED visit is sensitive to analytical assumptions and approaches. One potential explanation could be that the ED visit measure includes both emergent and non-emergent visits, with RPM likely having differential effects on the two ED types. Future studies could disaggregate ED visits to emergent and non-emergent and estimate the effect of RPM separately. Furthermore, access to and utilization patterns of ED could also affect the effectiveness of RPM.

Our finding of higher outpatient visits among RPM users is likely a consequence of regular follow-up built into the RPM modality for monthly reimbursements.^43^ Previous literature has shown that more physician office visits led to better blood pressure control.^44^ Likewise, patients who accessed patient portal systems had substantially higher outpatient visits and lower preventable hospitalizations,^45^ indicating that more interactions with the healthcare system have positive effects, particularly for older patients with comorbid conditions. Therefore, the effect of RPM use on mortality and hospitalizations may also be mediated through higher outpatient follow-up.

Our study should be interpreted in the context of its limitations. First, unmeasured confounding cannot be ruled out, as we do not have information on patients’ functional status, vital signs, access to healthcare, and clinicians’ preferences for RPM. RPM users could have had better access to healthcare services, and the lower mortality risk and higher outpatient visits could be an artifact of that access. Although we balanced baseline healthcare cost and utilization, the huge difference in baseline outpatient visits in the unmatched sample suggests that some dimensions of access to care may not have been balanced. Second, we could not evaluate the outcomes over a longer follow-up period, given that nearly 77% of the RPM users were from 2020. Third, we do not have information on who were offered RPM but denied it. Fourth, we could not study the frequency of RPM readings transmitted and monitored by the clinicians. Fifth, we did not consider the costs of RPM and RPM-related utilization, thereby not being able to answer whether RPM could be cost-saving, cost-effective, or neither. Sixth, as the study population is exclusively patients ≥65 years with higher rates of mortality and hospitalizations, the generalizability of these findings to younger population is uncertain.

In conclusion, we found meaningful reductions in hospitalizations and mortality, and substantial increases in outpatient visits with RPM use over a maximum follow-up of 6 months. Although the caveats of causal inference from retrospective, observational studies apply, these findings could inform expansion of RPM and the reimbursement decisions around it. Continuous evaluation of effectiveness of RPM services for chronic conditions with longer follow-up is warranted along with its potential cost-effectiveness and budget impact for public and private health systems.

### Supplementary Information:

Below is the link to the electronic supplementary material.

Supplementary file1 (DOCX 1196 KB)

## Data Availability

The Medicare fee-for-service data used for this study was purchased from Research Data Assistance Center (ResDAC). Due to the nature of Data Use Agreement (DUA), the data cannot be shared publicly nor with other researchers. Researchers interested in accessing the data used for this study can submit a DUA to the ResDAC’s Data Request Center.
