# Supplementary material for: Association of Remote Patient Monitoring with Mortality and Healthcare Utilization in Hypertensive Patients: a Medicare Claims–Based Study
Source: J Gen Intern Med. 2023 Nov 16;39(5):762–73. doi: 10.1007/s11606-023-08511-x (PMC11043264; doi:10.1007/s11606-023-08511-x)

**Supplement Tables and Figures**

**Supplement Table 1: Identification of clinical characteristics using diagnosis, procedure and drug codes**

| **Characteristics** | **Claims-based identification** |
| --- | --- |
| Remote patient monitoring | CPT: '99091', '99453', '99454', '99457', '99458', '99473', '99474' OR  CPT: '93229', '93268', '93270', '93271', '93272' AND CPT modifier ‘95’ |
| Hypertension, uncomplicated | ICD-10-CM: 'I10' |
| Hypertension, complicated | ICD-10-CM: 'I11', 'I12', 'I13', 'I15' |
| Cardiovascular-related hospitalization (conditions included: hypertension, hypertensive heart disease, hypertensive chronic kidney disease, ischemic heart disease, cardiac arrhythmias, heart failure, and stroke) | Admission or primary diagnosis variables with:  ICD-10-CM: 'I10', 'I11', 'I12', 'I13', 'I14', 'I15', 'I16', 'I20', 'I21', 'I22', 'I23', 'I24', 'I25', 'I30', 'I31', 'I32', 'I33', 'I34', 'I35', 'I36', 'I37', 'I38', 'I39', 'I40', 'I41', 'I42', 'I43', 'I44', 'I45', 'I46', 'I47', 'I48', 'I49', 'I50', 'I51', 'I52', 'I619', 'I63', 'I64', 'I739', 'I802', 'I959', 'R074', 'R071', 'G45', 'H341', 'I60', 'I61', 'I63', 'I64' |
| Any ED visit | CPT: '99281', '99282', '99283', '99284', '99285', '99291', '99292', '99234', '99235', '99236', '99217', '99218', '99219', '99220', '99224', '99225', '99226' AND  Place of service: ‘23’ |
| Cardiovascular-related ED visit | Any ED visit=1 AND  a diagnosis in the same claim for:  ICD-10-CM: 'I10', 'I11', 'I12', 'I13', 'I14', 'I15', 'I16', 'I20', 'I21', 'I22', 'I23', 'I24', 'I25', 'I30', 'I31', 'I32', 'I33', 'I34', 'I35', 'I36', 'I37', 'I38', 'I39', 'I40', 'I41', 'I42', 'I43', 'I44', 'I45', 'I46', 'I47', 'I48', 'I49', 'I50', 'I51', 'I52', 'I619', 'I63', 'I64', 'I739', 'I802', 'I959', 'R074', 'R071', 'G45', 'H341', 'I60', 'I61', 'I63', 'I64' |
| Any outpatient visit | Place of service: ‘11’, ‘19’, ‘22’ |
| Cardiovascular-related outpatient visit | Any outpatient visit=1 AND  a diagnosis in the same claim for:  ICD-10-CM: 'I10', 'I11', 'I12', 'I13', 'I14', 'I15', 'I16', 'I20', 'I21', 'I22', 'I23', 'I24', 'I25', 'I30', 'I31', 'I32', 'I33', 'I34', 'I35', 'I36', 'I37', 'I38', 'I39', 'I40', 'I41', 'I42', 'I43', 'I44', 'I45', 'I46', 'I47', 'I48', 'I49', 'I50', 'I51', 'I52', 'I619', 'I63', 'I64', 'I739', 'I802', 'I959', 'R074', 'R071', 'G45', 'H341', 'I60', 'I61', 'I63', 'I64' |
| Myocardial infarction | ICD-10-CM: 'I21', 'I22' |
| Congestive heart failure | ICD-10-CM: 'I099', 'I110', 'I130', 'I132', 'I255', 'I420', 'I425', 'I426', 'I427', 'I428', 'I429', 'I43', 'I50', 'P290' |
| Cardiac arrhythmia | ICD-10-CM: I47', 'I48', 'I49', 'I441', 'I442', 'I443', 'I456', 'I459', 'R000', 'R001', 'R008', 'T821', 'Z450', 'Z950' |
| Valvular disease | ICD-10-CM: 'I70', 'I71', 'I731', 'I738', 'I739', 'I771', 'I790', 'I792', 'K551', 'K558', 'K559', 'Z958', 'Z959' |
| Circulation disorders | ICD-10-CM: 'I26', 'I27', 'I280', 'I288', 'I289' |
| Peripheral vascular disorders | ICD-10-CM: 'I70', 'I71', 'I731', 'I738', 'I739', 'I771', 'I790', 'I792', 'K551', 'K558', 'K559', 'Z958', 'Z959' |
| Paralysis | ICD-10-CM: 'G81', 'G82', 'G041', 'G114', 'G801', 'G802', 'G830', 'G831', 'G832', 'G833', 'G834', 'G839' |
| Other Neurological disorders | ICD-10-CM: 'G10', 'G11', 'G12', 'G13', 'G20', 'G21', 'G22', 'G32', 'G35', 'G36', 'G37', 'G40', 'G41', 'R56', 'G254', 'G255', 'G312', 'G318', 'G319', 'G931', 'G934', 'R470' |
| Chronic pulmonary disease | ICD-10-CM: 'I278', 'I279', 'J684', 'J701', 'J703', 'J40', 'J41', 'J42', 'J43', 'J44', 'J45', 'J46', 'J47', 'J60', 'J61', 'J62', 'J63', 'J64', 'J65', 'J66', 'J67' |
| Diabetes, uncomplicated | ICD-10-CM: 'E100', 'E101', 'E109', 'E110', 'E111', 'E119', 'E120', 'E121', 'E129', 'E130', 'E131', 'E139', 'E140', 'E141', 'E149' |
| Diabetes, complicated | ICD-10-CM: 'E102', 'E103', 'E104', 'E105', 'E106', 'E107', 'E108', 'E112', 'E113', 'E114', 'E115', 'E116', 'E117', 'E118', 'E122', 'E123', 'E124', 'E125', 'E126', 'E127', 'E128', 'E132', 'E133', 'E134', 'E135', 'E136', 'E137', 'E138', 'E142', 'E143', 'E144', 'E145', 'E146', 'E147', 'E148' |
| Hypothyroidism | ICD-10-CM: 'E00', 'E01', 'E02', 'E03', 'E890' |
| Renal failure | ICD-10-CM: 'N18', 'N19', 'I120', 'I131', 'N250', 'Z490', 'Z491', 'Z492', 'Z940', 'Z992' |
| Liver disease | ICD-10-CM: 'B18', 'I85', 'K70', 'K72', 'K73', 'K74', 'I864', 'I982', 'K711', 'K713', 'K714', 'K715', 'K717', 'K760', 'K762','K763','K764','K765','K766','K767','K768','K769','Z944' |
| Peptic ulcer disease | ICD-10-CM: 'K257', 'K259', 'K267', 'K269', 'K277', 'K279', 'K287', 'K289' |
| HIV/AIDS | ICD-10-CM: 'B20', 'B21', 'B22', 'B24' |
| Lymphoma | ICD-10-CM: 'C81', 'C82', 'C83', 'C84', 'C85', 'C88', 'C96', 'C900', 'C902' |
| Solid tumor without metastasis | ICD-10-CM: 'C00', 'C01', 'C02', 'C03', 'C04', 'C05', 'C06', 'C07', 'C08', 'C09', 'C10', 'C11', 'C12', 'C13', 'C14', 'C15', 'C16', 'C17', 'C18', 'C19', 'C20', 'C21', 'C22', 'C23', 'C24', 'C25', 'C26', 'C30', 'C31', 'C32', 'C33', 'C34', 'C37', 'C38', 'C39', 'C40', 'C41', 'C43', 'C45', 'C46', 'C47', 'C48', 'C49', 'C50', 'C51', 'C52', 'C53', 'C54', 'C55', 'C56', 'C57', 'C58', 'C60', 'C61', 'C62', 'C63', 'C64', 'C65', 'C66', 'C67', 'C68', 'C69', 'C70', 'C71', 'C72', 'C73', 'C74', 'C75', 'C76', 'C97' |
| Metastatic cancer | ICD-10-CM: 'C77', 'C78', 'C79', 'C80' |
| Rheumatoid arthritis | ICD-10-CM: 'M05', 'M06', 'M08', 'M30', 'M32', 'M33', 'M34', 'M35', 'M45', 'L940', 'L941', 'L943', 'M120', 'M123', 'M310', 'M311', 'M312', 'M313', 'M461', 'M468', 'M469' |
| Coagulopathy | ICD-10-CM: 'D65', 'D66', 'D67', 'D68', 'D691', 'D693', 'D694', 'D695', 'D696' |
| Obesity | ICD-10-CM: 'E66' |
| Weight loss | ICD-10-CM: 'E40', 'E41', 'E42', 'E43', 'E44', 'E45', 'E46', 'R64', 'R634' |
| Fluid and electrolyte disorders | ICD-10-CM: 'E86', 'E87', 'E222' |
| Blood loss anemia | ICD-10-CM: 'D500' |
| Iron deficiency anemia | ICD-10-CM: 'D51','D52','D53', 'D508','D509' |
| Alcohol abuse | ICD-10-CM: 'F10', 'E52', 'T51', 'G621', 'I426', 'K292', 'K700', 'K703', 'K709', 'Z502', 'Z714', 'Z721' |
| Drug abuse | ICD-10-CM: 'F11', 'F12', 'F13', 'F14', 'F15', 'F16', 'F18', 'F19', 'Z715', 'Z722' |
| Psychoses | ICD-10-CM: 'F20', 'F22', 'F23', 'F24', 'F25', 'F28', 'F29', 'F302', 'F312', 'F315' |
| Depression | ICD-10-CM: 'F32', 'F33', 'F204', 'F313', 'F314', 'F315', 'F341', 'F412', 'F432' |
| Sleep disorders | ICD-10-CM: 'G47' |
| Multiple sclerosis | ICD-10-CM: 'G45' |
| Pneumonia | ICD-10-CM: 'J120', 'J121', 'J122', 'J1281', 'J123', 'J1289', 'J129', 'J13', 'J181', 'J150', 'J151', 'J14', 'J154', 'J153', 'J1520', 'J15211', 'J15212', 'J1529', 'J158', 'J155', 'J156', 'A481', 'J159', 'J157', 'J160', 'J168', 'B250', 'A3701', 'A3711', 'A3781', 'A3791', 'A221', 'B440', 'J17', 'B7781', 'J180', 'J188', 'J189', 'J1000', 'J1001', 'J1008', 'J1100', 'J1108', 'J129' |
| Septicemia | ICD-10-CM: 'A021', 'A207', 'A227', 'A312', 'A392', 'A393', 'A394', 'A400', 'A401', 'A403', 'A408', 'A409', 'A412', 'A4101', 'A4102', 'A411', 'A403', 'A414', 'A4150', 'A413', 'A4151', 'A4152', 'A4153', 'A4159', 'A4181', 'A4189', 'A427', 'A267', 'A327', 'A5486', 'B377', 'A419', 'B007', 'R7881', 'A021', 'A227', 'A267', 'A327', 'A400', 'A401', 'A403', 'A408', 'A409', 'A4101', 'A4102', 'A411', 'A412', 'A413', 'A414', 'A4150', 'A4151', 'A4152', 'A4153', 'A4159', 'A4181', 'A4189', 'A419', 'A427', 'A5486', 'B377', 'R6520', 'R6521' |
| Stroke | ICD-10-CM: 'G45', 'H341', 'I60', 'I61', 'I63', 'I64' |
| Annual wellness visits | HCPCS: 'G0438', 'G0439' |
| Flu vaccination | HCPCS: '90630', '90653', '90654', '90655', '90656', '90657', '90661', '90662', '90672', '90673', '90674', '90682', '90685', '90686', '90687', '90688', '90756', 'Q2035', 'Q2037', 'Q2038' |
| Chronic care management | HCPCS: '99487', '99489', '99490', '99491', 'G0506' |

**Supplement Figure 1: Distribution of propensity scores in the overall sample**


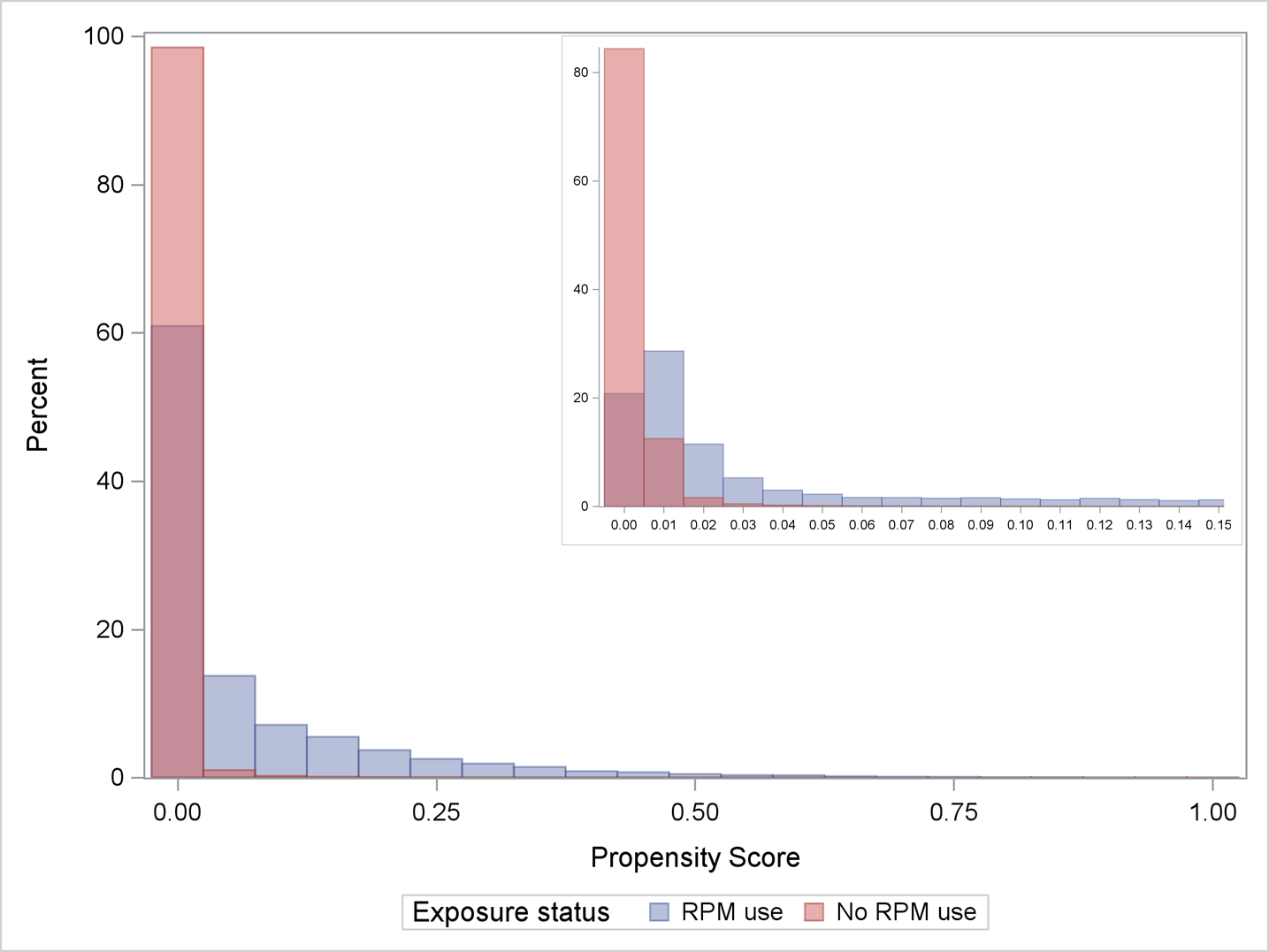
 Note: The inset chart is included to increase visibility and represents the same distribution of propensity scores but for focusing on the scores between 0 and 0.15.

**Supplement Figure 2: Distribution of propensity scores in the matched sample**


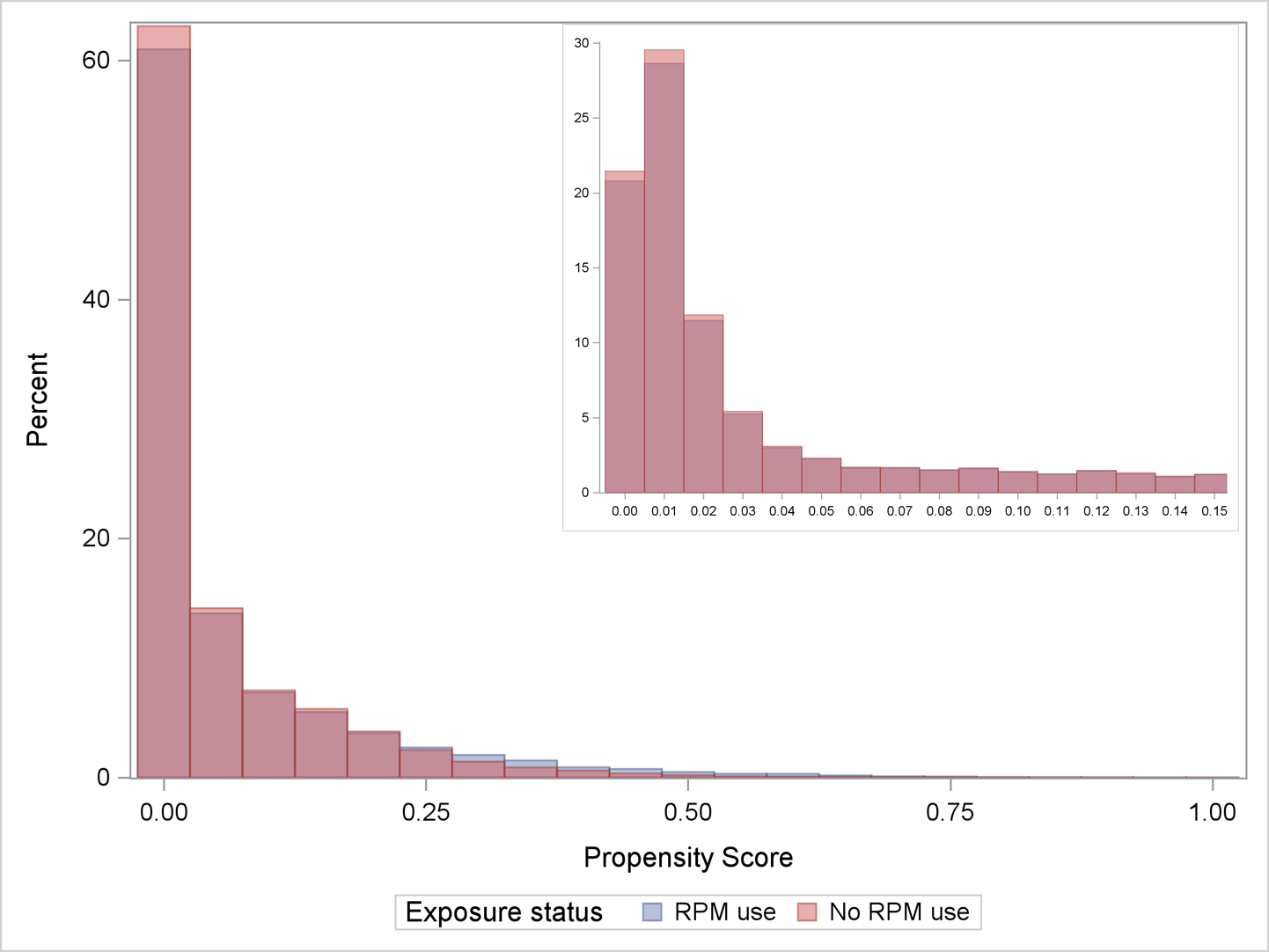
 Note: The inset chart is included to increase visibility and represents the same distribution of propensity scores but for focusing on the scores between 0 and 0.15.

**Supplement Table 2: Number and percentages of patients with multiple outcomes**

| **Outcomes** | **RPM users (n=16,339); n (%)** | **RPM non-users (n=63,333); n (%)** |
| --- | --- | --- |
| All-cause mortality only | 44 (0.27) | 340 (0.54) |
| Any hospitalization only | 0 (0.00) | 33 (0.05) |
| Any ED visit only | 17 (0.10) | 172 (0.27) |
| Any outpatient visit only | 12,331 (75.47) | 44,120 (69.66) |
| Hospitalization and mortality | 2 (0.01) | 42 (0.07) |
| ED visit and mortality | 3 (0.02) | 51 (0.08) |
| Outpatient visit and mortality | 65 (0.40) | 366 (0.58) |
| Hospitalization, ED visit and mortality | 33 (0.20) | 184 (0.29) |
| Hospitalization, outpatient visit and mortality | 11 (0.07) | 120 (0.19) |
| ED visit, outpatient visit and mortality | 32 (0.20) | 155 (0.24) |
| Hospitalization, ED visit, outpatient visit and mortality | 240 (1.47) | 1,261 (1.99) |
| Hospitalization and ED visit | 30 (0.18) | 121 (0.19) |
| Hospitalization and outpatient visit | 267 (1.63) | 2,121 (3.35) |
| ED visit and outpatient visit | 1,535 (9.39) | 5,474 (8.64) |
| Hospitalization, ED visit and outpatient visit | 1,443 (8.83) | 5,955 (9.40) |

**Supplement Figure 3: Kaplan Meier curves for any hospitalization outcome in the matched sample in the primary analysis**


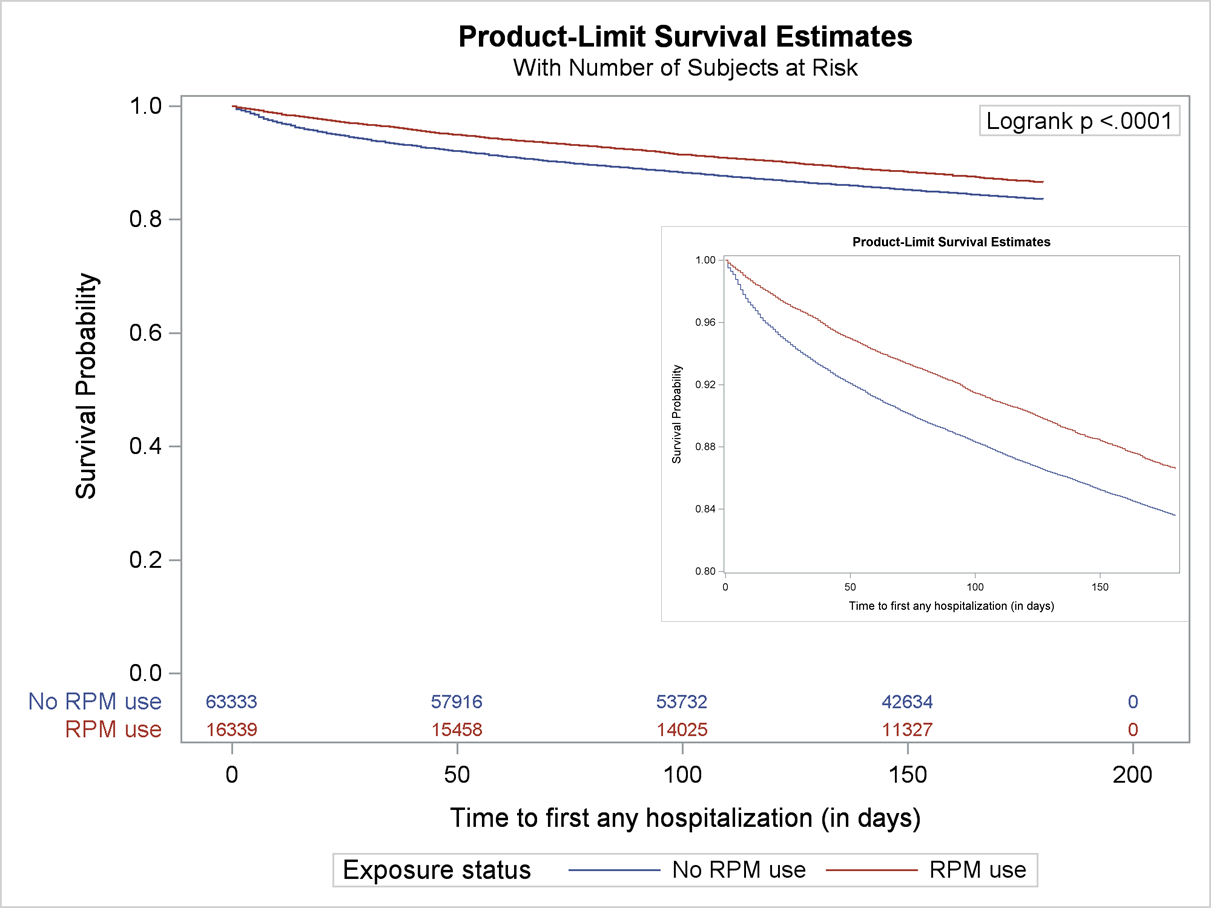
 Note: The inset chart is included to increase visibility and represents the same Kaplan-Meier curves for any hospitalization outcome with the Y-axis beginning at 0.80 survival probability.

**Supplement Figure 4: Kaplan Meier curves for cardiovascular-related hospitalization outcome in the matched sample in the primary analysis**


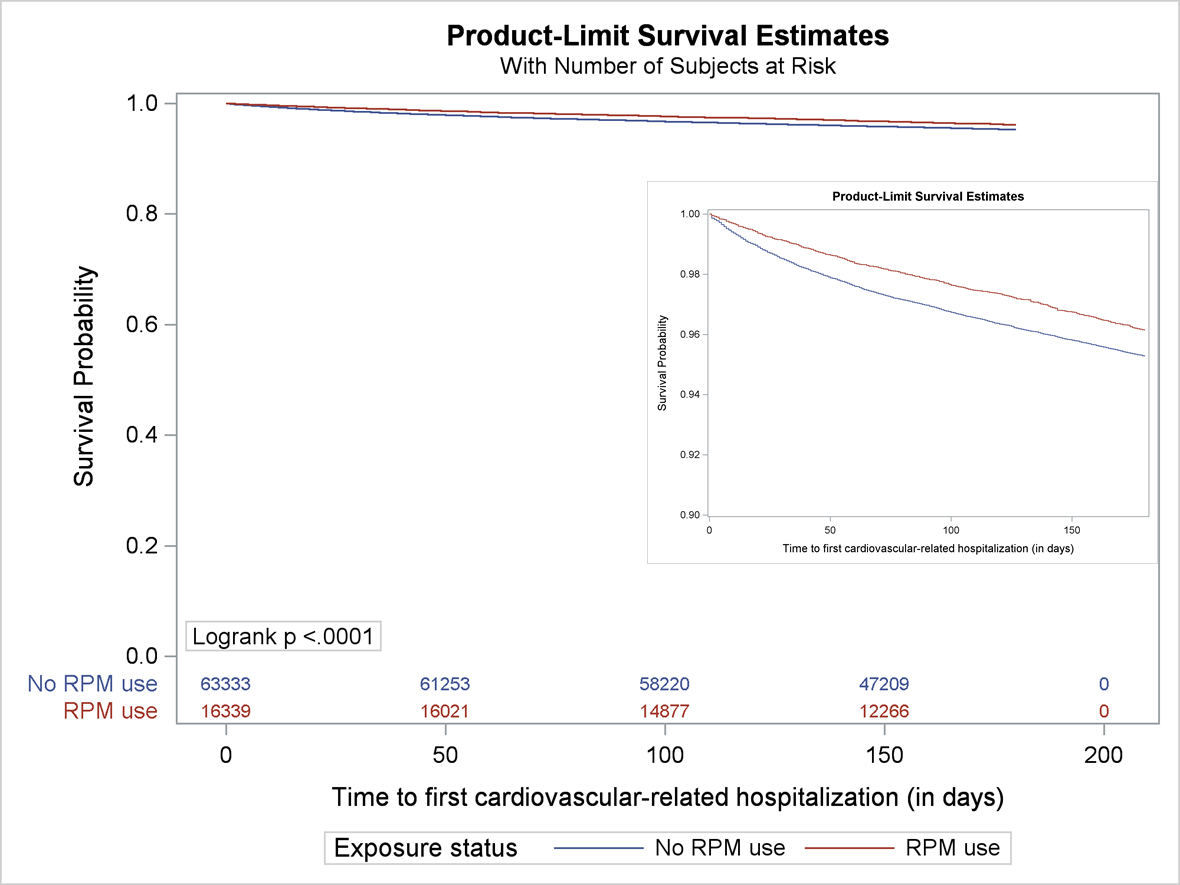
 Note: The inset chart is included to increase visibility and represents the same Kaplan-Meier curves for cardiovascular-related hospitalization outcome with the Y-axis beginning at 0.90 survival probability.

**Supplement Figure 5: Kaplan Meier curves for non-cardiovascular-related hospitalizations in the matched sample in the primary analysis**


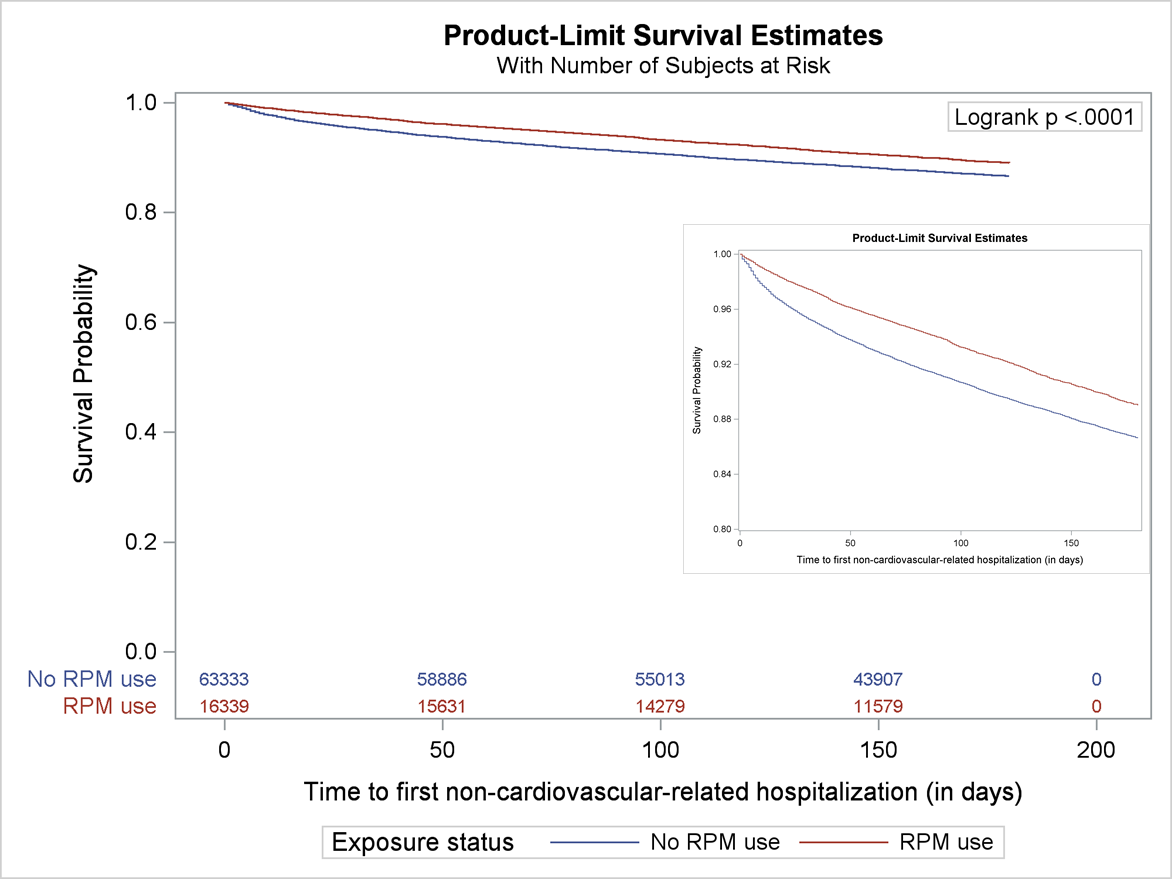
 Note: The inset chart is included to increase visibility and represents the same Kaplan-Meier curves for non-cardiovascular-related hospitalization outcome with the Y-axis beginning at 0.80 survival probability.

**Supplement Figure 6: Kaplan Meier curves for any Emergency Department (ED) visit in the matched sample in the primary analysis**


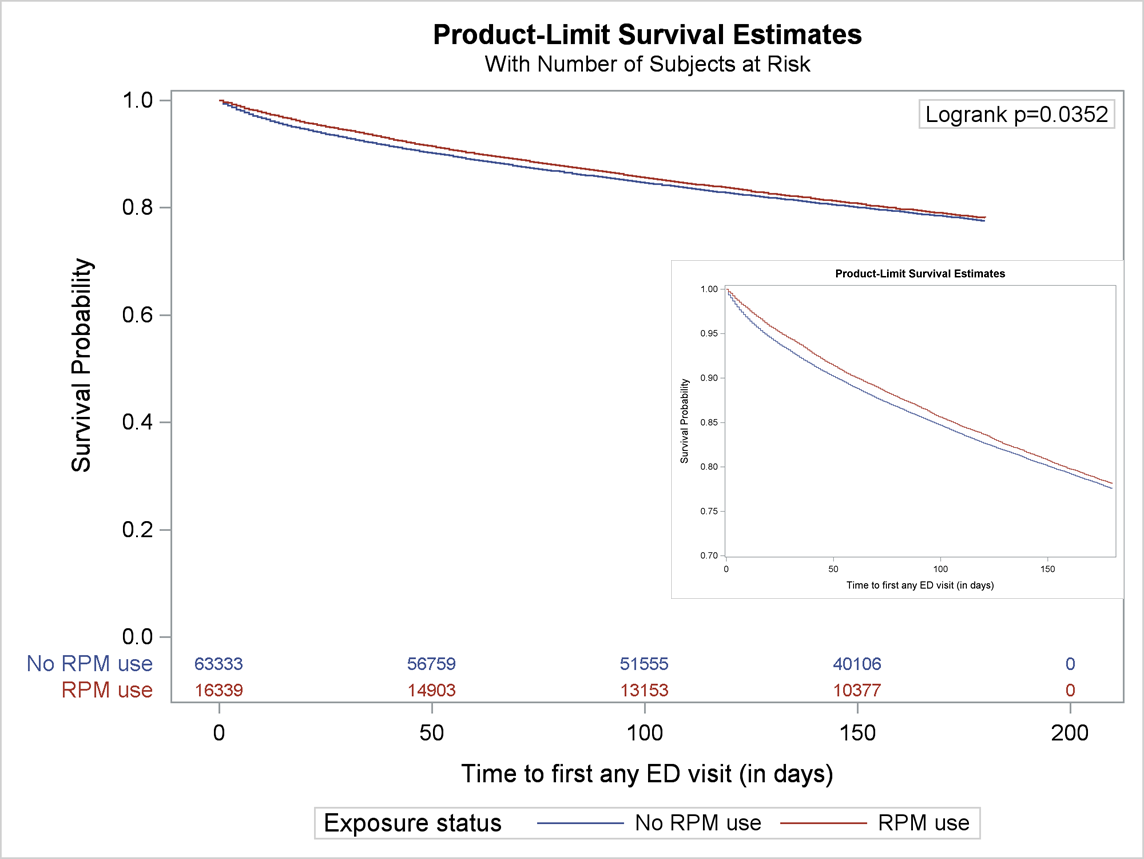
 Note: The inset chart is included to increase visibility and represents the same Kaplan-Meier curves for any ED visit outcome with the Y-axis beginning at 0.70 survival probability.

**Supplement Figure 7: Kaplan Meier curves for cardiovascular-related Emergency Department (ED) visit in the matched sample in the primary analysis**


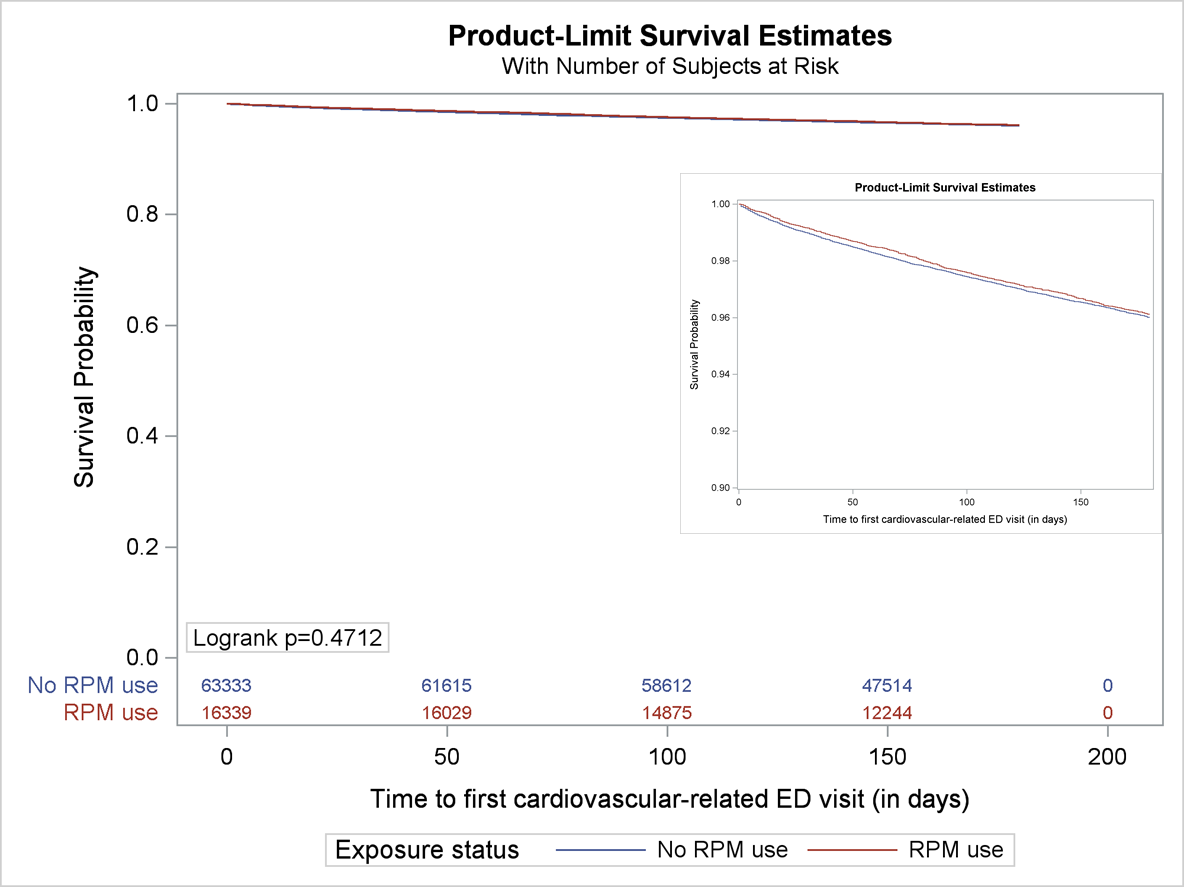
 Note: The inset chart is included to increase visibility and represents the same Kaplan-Meier curves for cardiovascular-related ED visit outcome with the Y-axis beginning at 0.90 survival probability.

**Supplement Figure 8: Kaplan Meier curves for non-cardiovascular-related Emergency Department (ED) visit in the matched sample in the primary analysis**


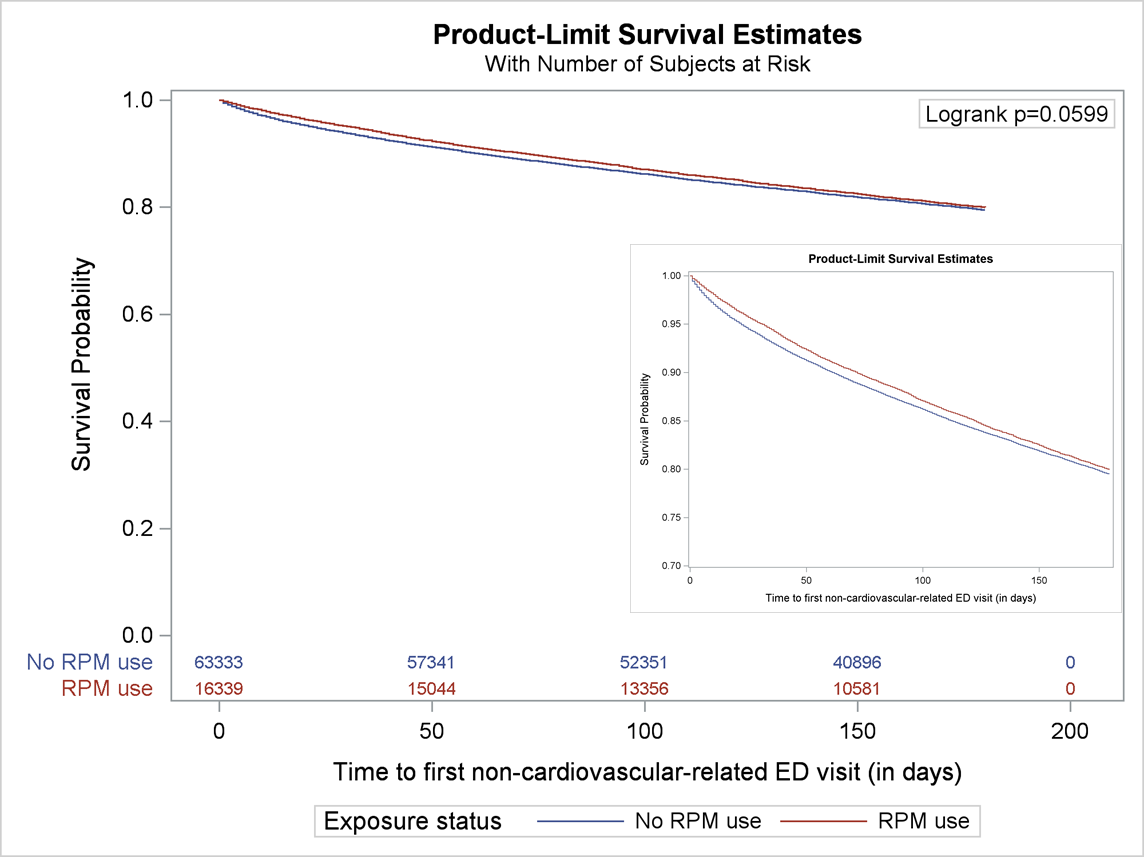
 Note: The inset chart is included to increase visibility and represents the same Kaplan-Meier curves for non-cardiovascular-related ED visit outcome with the Y-axis beginning at 0.70 survival probability.

**Supplement Figure 9: Kaplan Meier curves for any outpatient visit in the matched sample in the primary analysis**


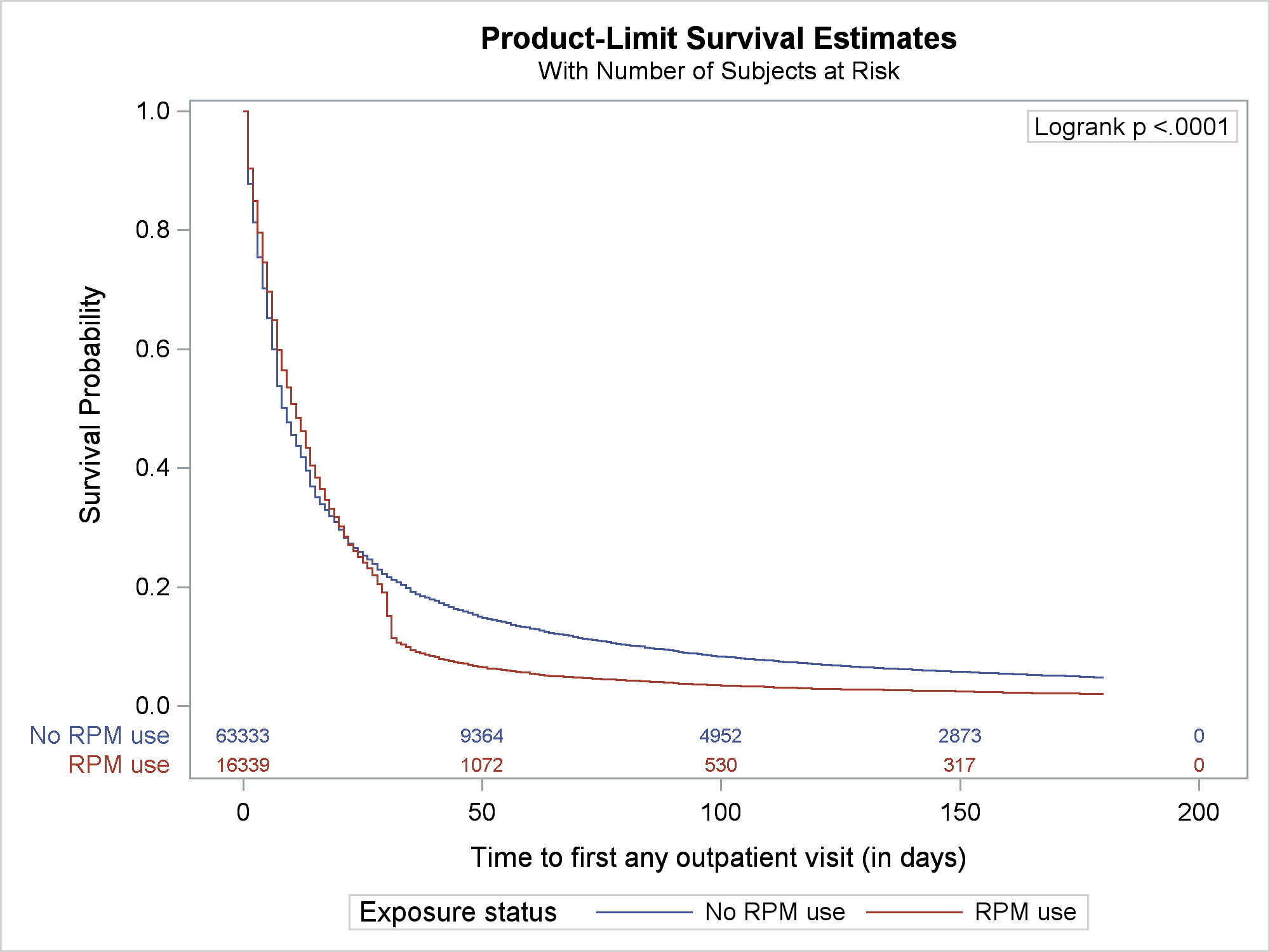


**Supplement Figure 10: Kaplan Meier curves for cardiovascular-related outpatient visit in the matched sample in the primary analysis**

**
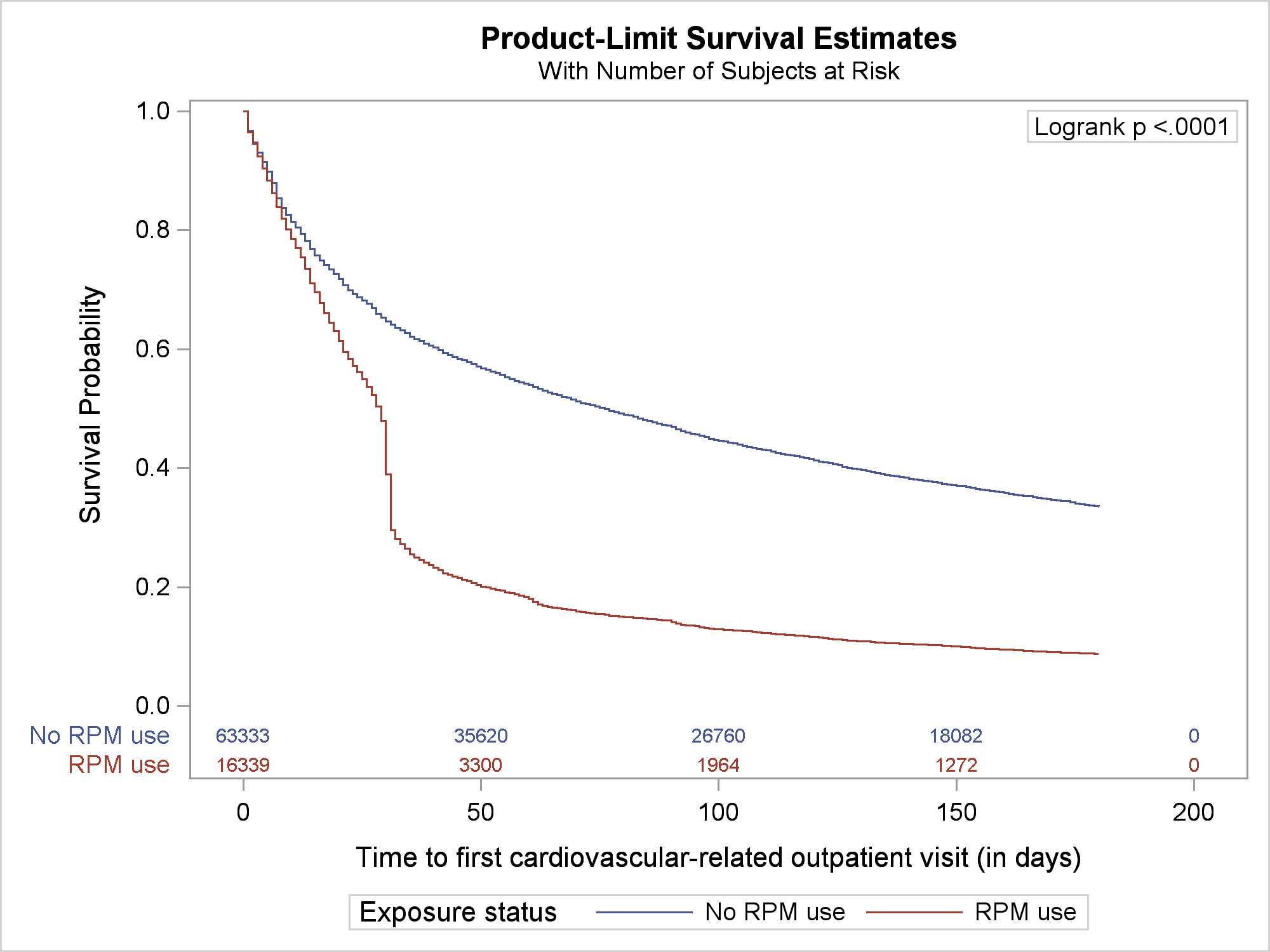
**

**Supplement Figure 11: Kaplan Meier curves for non-cardiovascular-related outpatient visit in the matched sample in the primary analysis**


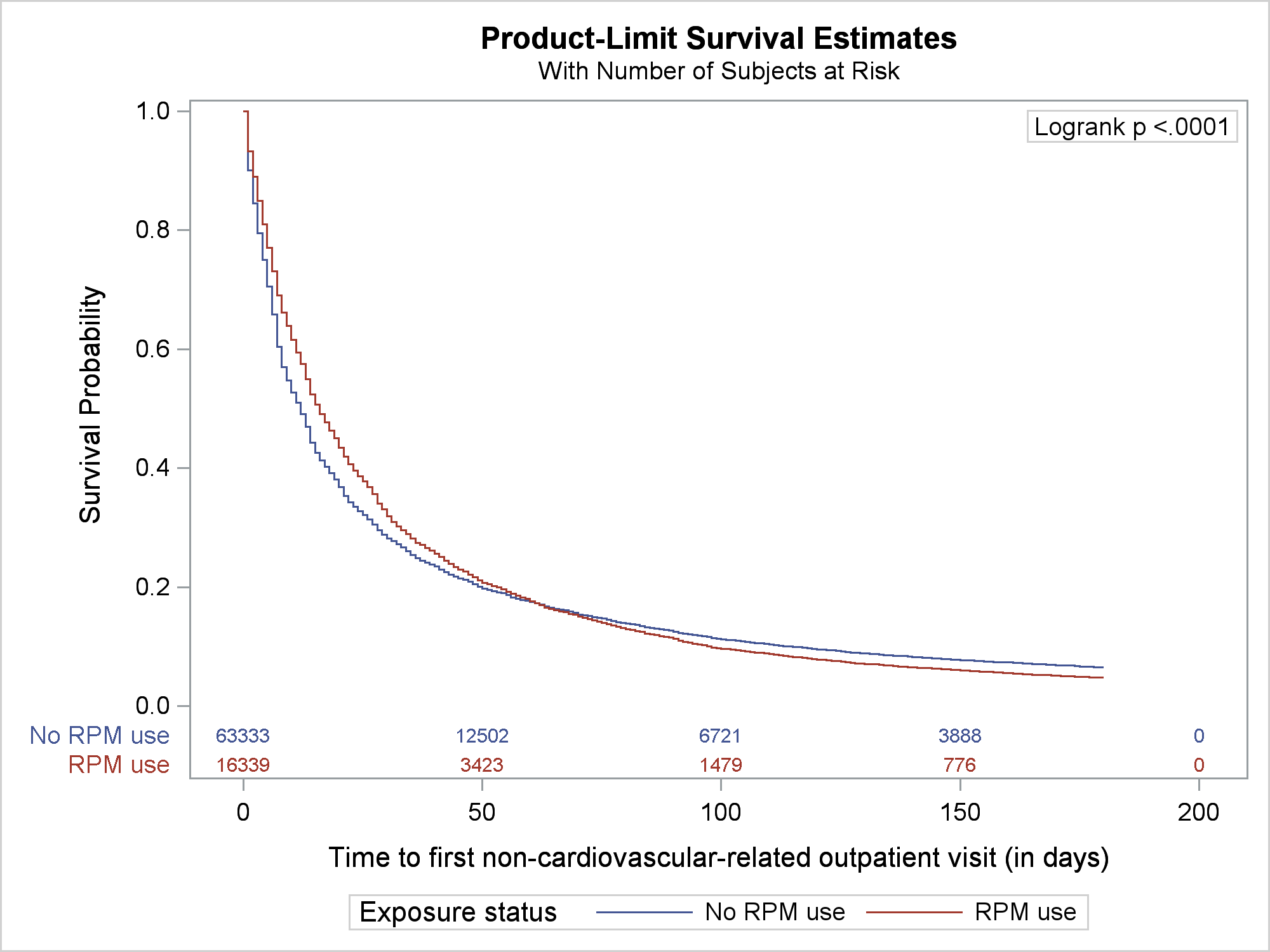

Supplement: Supplementary file 1 — Supplementary file1 (DOCX 1196 KB) [file 11606_2023_8511_MOESM1_ESM.docx]
